# Effect of a Plant-Based vs Omnivorous Soul Food Diet on Weight and Lipid Levels Among African American Adults: A Randomized Clinical Trial

**DOI:** 10.1001/jamanetworkopen.2022.50626

**Published:** 2023-01-12

**Authors:** Gabrielle M. Turner-McGrievy, Sara Wilcox, Edward A. Frongillo, E. Angela Murphy, Brent Hutto, Mary Wilson, Marty Davey, John A. Bernhart, Nkechi Okpara, Shiba Bailey, Emily Hu

**Affiliations:** 1 Department of Health Promotion, Education and Behavior, Arnold School of Public Health, University of South Carolina, Columbia; 2 Prevention Research Center, Arnold School of Public Health, University of South Carolina, Columbia; 3 Department of Exercise Science, Arnold School of Public Health, University of South Carolina, Columbia; 4 School of Medicine, University of South Carolina, Columbia; 5 Department of Health Services, Policy, and Management, Arnold School of Public Health, University of South Carolina, Columbia

## Abstract

**Importance:**

More African American individuals die from cardiovascular disease (CVD) than any other chronic disease condition. Despite this disparity, African American individuals are underrepresented in nutrition and CVD interventions.

**Objective:**

To compare the effects of an entirely plant-based (vegan) or low-fat omnivorous (omni) diet on change in body weight and lipids during a 2-year intervention.

**Design, Setting, and Participants:**

The Nutritious Eating With Soul (NEW Soul) study was a 2-year, randomized clinical trial conducted in 2 cohorts (2018-2020 and 2019-2021) that took place in a university teaching kitchen in Columbia, South Carolina (before March 2020), and via online videoconference sessions (after March 2020). Participants included African American adults aged 18 to 65 years with overweight or obesity (body mass index of 25.0-49.9) and without type 2 diabetes, uncontrolled thyroid disease, recent weight loss, or pregnancy. Data assessors and statisticians were blinded to study condition. Data analysis was performed from March to June 2022.

**Interventions:**

The intervention included weekly nutrition classes for 6 months biweekly classes for 6 months, and monthly classes for 12 months. Dietary interventions either emphasized no animal product intake (vegan) or a low-fat omnivorous diet (omni). Both dietary patterns emphasized soul food cuisine (traditional African American southern foodways).

**Main Outcomes and Measures:**

Primary outcomes included change in body weight and lipid measures at 12 months.

**Results:**

There were 568 participants who completed an online screening questionnaire; 409 were excluded and 159 were randomized (77 to the vegan group and 82 to the omni group). Of the 159 participants (mean [SD] age, 48.4 [10.6] years; 126 female [79%]) who began the study, the main outcome of body weight was obtained for 121 participants (76%) at 12 months. There were no differences in outcomes between groups, including 12-month changes in weight (mean, –2.39 kg [95% CI, –3.48 to –1.30 kg] for the vegan group vs –2.03 kg [95% CI, –3.07 to –1.00 kg] for the omni group; *P* = .64), total cholesterol (–1.05 mg/dL [95% CI, –9.60 to 7.50 mg/dL] for the vegan group vs 1.66 mg/dL [95% CI, –7.20 to 10.50 mg/dL] for the omni group; *P* = .67), or low-density lipoprotein cholesterol (mean, –2.56 mg/dL [95% CI, –9.52 to 4.40 mg/dL] for the vegan group vs –0.79 mg/dL [95% CI, –7.98 to 6.40 mg/dL] for the omni group; *P* = .73). Weight loss at 12 months among cohort 1, whose weight was assessed in 2019 before the COVID-19 pandemic, was significantly greater than that for cohort 2, whose weight was assessed summer 2020 during COVID-19 (–3.45 kg [95% CI, –4.67 to –2.22 kg] vs –1.24 kg [95% CI, –2.24 to –0.25 kg]; *P* = .01).

**Conclusions and Relevance:**

In this randomized clinical trial examining weight loss and CVD risk factor reduction among African American adults, there were no differences between the groups, and the magnitude of changes overall was small.

**Trial Registration:**

ClinicalTrials.gov Identifier: NCT03354377

## Key Points

**Question:**

What are the impacts of an entirely plant-based vs a low-fat, reduced–animal product omnivorous diet on changes in 12-month and 24-month body weight and lipids among African American adults at risk for cardiovascular disease (CVD)?

**Findings:**

In this randomized clinical trial of 159 adults, participants in both groups saw similar improvements in body weight and CVD risk factor outcomes.

**Meaning:**

Both plant-based and low-fat omnivorous soul food diets produced modest weight loss and CVD risk-related improvements among African American adults.

## Teaser

This randomized clinical trial compares the effects of an entirely plant-based vegan or low-fat omnivorous diet on change in body weight and lipids during a 2-year intervention.

## Introduction

In the US, African American individuals die from cardiovascular disease (CVD) more than any other chronic disease.^1^ Overweight and obesity are associated with type 2 diabetes,^2^ hypertension, and CVD.^3^ Despite higher CVD rates, African American individuals are underrepresented in nutrition and CVD interventions.^4^

Although several studies^5,6^ have found evidence suggesting that plant-based diets (PBDs) may be protective against CVD, most have been observational. The few interventions examining PBDs have found that they have the potential to reduce factors associated with risk of CVD, such as trimethylamine-N-oxide,^7^ C-reactive protein,^8^ and low-density lipoprotein (LDL) cholesterol.^9^ African American vegetarians and vegans have significantly lower risks of hypertension, diabetes, and elevated cholesterol than African American omnivores.^10^ PBDs have increased in popularity among African American individuals over the past decade. Approximately 8% of African American individuals follow a vegan diet compared with 3% of all US individuals.^11^ Vegan soul food has emerged as a popular cuisine, particularly in the southeastern US.^12^ Traditional soul food cuisine emphasizes both animal-based (eg, fried chicken) and vegetable-based (eg, black-eyed peas and collard greens) foods. Vegan soul food has a focus solely on plant-based takes on traditional foods.^13^

The Nutritious Eating With Soul (NEW Soul) study, a 2-year behavioral nutrition intervention among African American individuals with overweight or obesity, was conducted to address the limited inclusion of African American individuals in nutrition interventions and limited prior intervention work examining the impact of a PBD on CVD risk factors. The intervention compared 2 diets, both emphasizing soul food cuisine: entirely PBD (vegan) or low-fat omnivorous (omni). The aim of this study was to compare the effects of each diet on the primary outcomes of change in body weight and lipids across the 2-year study, with the primary outcomes examined at 12 months and maintenance examined at 24 months.^14^ A post hoc secondary aim was to examine the difference in weight loss at 12 months between cohort 1 (who had their weight assessed before the COVID-19 pandemic) and cohort 2 (who had their weight assessed after the onset of the COVID-19 pandemic). We hypothesized that the vegan group would have greater improvements in body weight and lipids compared with the omni group and, as a post hoc hypothesis, that cohort 1 would lose significantly more weight at 12 months than cohort 2.

## Methods

### Study Participants

The NEW Soul intervention, inclusion criteria, and measures have been described elsewhere.^15^ Briefly, NEW Soul was a single-site, parallel-group, 2-year intervention among participants aged 18 to 65 years with overweight or obesity (defined as body mass index [BMI; calculated as weight in kilograms divided by height in meters squared] of 25.0-49.9) who self-identify as African American. Participants were recruited in 2 cohorts separated by 1 year starting in 2018. Recruitment strategies are described elsewhere.^16^ Qualified participants attended an orientation session, where they signed a consent form and completed questionnaires and 1 dietary recall in a computer laboratory. Participants were randomized in blocks of 10 (with blocks created using a computerized random-number generator) and were stratified by sex to ensure equal distribution of men and women between groups. Once all baseline data were collected, a blinded study coordinator (M.W.) assigned participants to the next number in the block. Participants did not learn of their randomized assignment until they attended their first group meeting. The study was approved by the University of South Carolina institutional review board and was overseen by a data safety and monitoring board. Participants received incentives for completing all measures ($10 at 3 months, $50 at 6 months, $50 at 12 months, and $100 at 24 months). The Consolidated Standards of Reporting Trials (CONSORT) reporting guideline for randomized clinical trials was used for this study. See the trial protocol for more information (Supplement 1).

### Measures

Measures for the NEW Soul intervention have been described elsewhere.^15,17,18,19^ Details of all measures are provided in Table 1. Primary outcomes for the study included change in body weight and LDL cholesterol at 12 months. All assessments were conducted by trained assessors who were blinded to study condition.

**Table 1.** Measures Assessed and the Time Points in the Nutritious Eating With Soul Study

Measurement target	Measurement method	Assessment time				
		Baseline	3 mo	6 mo	12 mo	24 mo
Anthropometric and body composition data						
Height	Wall-mounted stadiometer (Model S100, Ayerton Corp)	Yes	No	No	No	No
Weight	Assessed with participants wearing light street clothes using a calibrated digital scale (Healthometer model 500 KL)	Yes	Yes	Yes	Yes	Yes
Waist and hip circumference	Using a spring-loaded tape measure, waist circumference was assessed at the iliac crest,^17^ and hip circumference was measured at the maximum protuberance of the buttocks^18^	Yes	No	Yes	No	No
Body fat (percentage) and lean (kilograms) mass of total body	Dual-energy x-ray absorptiometry scan was performed by a certified technician	Yes	No	No	Yes	No
Dietary intake						
Energy intake (kilocalories) and food groups	Three unannounced 24-h dietary recalls using the National Cancer Institute’s Automated Self-Administered 24-hour Dietary Recall (ASA24),^19^ including 1 weekend day (Friday-Sunday) and 2 weekdays	Yes	Yes	Yes	Yes	Yes
Cardiovascular disease risk factors						
Fasting lipid panel (total, low-density lipoprotein, and high-density lipoprotein cholesterol, and triglycerides), glucose, and insulin	All blood samples were performed on serum or plasma using commercially available assay kits; all samples from the same participant were analyzed on the same plate and, thus, under the same conditions	Yes	No	Yes	Yes	Yes
Blood pressure	After a 5-min rest period, blood pressure is assessed using an Omron Hem 705 CP Auto Inflate Blood Pressure Monitor; a minimum of 2 readings (and a maximum of 4) were taken, and the average of those readings is used	Yes	No	Yes	Yes	Yes

### Anthropometric and Body Composition Data

Participants who were unable to come to the study site were asked to provide a self-reported weight. These weights were not included in the final analyses, but the number of participants self-reporting weights are included in the CONSORT diagram to indicate continued engagement with the study (Supplement 1).

### Dietary and Questionnaire Data

Participants completed three 24-hour dietary recalls, which were averaged to create a mean energy intake at each time point (baseline and 3, 6, 12, and 24 months). Participants were also asked what medications they were currently taking. Dietary adherence was assessed by examining recommended animal product intake for 5 food groups via the averaged dietary recalls at each assessment time point. Participants received 1 point for each food group (omni: eggs, ≤0.3 servings per day; seafood, ≥0.3 servings per day; poultry, ≤1 serving [3 oz; 84 g] per day; red meat, ≤0.67 serving [2 oz; 56 g] per day; and dairy, ≥2 servings per day; vegan: received 1 point for 0 servings per day within each food group). Adherence scores were dichotomized such that scores of 2.5 or higher were considered mostly adherent.

### CVD Risk Factors

To assess changes in risk factors for CVD, blood pressure was taken along with a fasting lipid panel (total, LDL, and high-density lipoprotein cholesterol, and triglycerides), glucose, and insulin. Details on these procedures have been provided elsewhere.^15^

### Dietary and Behavioral Interventions

Apart from the diet prescribed, participants received similar behavioral interventions. Participants attended weekly group-based classes for 6 months, biweekly for 6 months, and monthly for 12 months. Participants were provided with additional electronic content: a website to access study materials and recordings of class sections, which they could view to make up a missed class; a private Facebook group starting at 6 months; and biweekly podcasts and newsletters starting at 12 months. Behavioral strategies for the classes were informed by Social Cognitive Theory^20^ (such as goal setting and targeting self-efficacy for healthy eating) and the Diabetes Prevention Program.^21^

The diets used in NEW Soul are described elsewhere,^15^ and both were informed by the Oldways African Heritage Diet^22^ and followed a healthy soul food diet plan. The vegan group was asked to consume a diet from whole plant foods (fruits, vegetables, legumes, whole grains, nuts, and seeds). Participants were asked to limit processed fats (eg, margarine) and oils in favor of whole plant fat sources, such as nuts, seeds, and avocados and avoid all meat, fish, poultry, eggs, and dairy. The omni group’s recommendations were based on the Therapeutic Lifestyle Change diet^23^ and recommended a diet reduced in animal product intake. This included limited meat (≤5 oz [140 g] of lean meat per day) and eggs (≤2 yolks per week) and emphasized low-fat dairy, fish, fruits, vegetables, legumes, and whole grains.^23^ The Therapeutic Lifestyle Change diet was chosen as the comparison diet because it is recommended by the National Institute of Health’s National Cholesterol Education Program as the dietary approach for lowering cholesterol levels.^23^ All classes included a review of the previous class’s SMART (Specific, Measurable, Achievable, Relevant, and Time-Bound) goal^24^; a discussion of successes and challenges from the previous week led by a community facilitator; a presentation of a nutrition topic (eg, greens, making a meal plan, and protein); a cooking demonstration by a community soul food chef, NEW Soul nutrition interventionist (M.D.), or hands-on in groups; a physical activity or stress management activity; and setting of the next SMART goal. Participants were provided with a binder with the food group recommendations, example meal ideas, and starter recipes. At each class, participants received additional handouts related to the topics covered in class and recipes. The difference between the groups was part of the nutrition content and foods prepared during the cooking demonstrations. On the basis of CONSERVE (CONSORT and SPIRIT Extension for RCTs Revised in Extenuating Circumstances) guidelines,^25,26,27,28,29,30^ alterations to interventions and assessments were made because of COVID-19 and are detailed in Table 2.^31^

**Table 2.** CONSERVE (CONSORT and SPIRIT Extension for Randomized Clinical Trials Revised in Extenuating Circumstances) Detailing the Alterations Made to Intervention and Assessments Based on COVID-19

CONSERVE reporting categories	Details of each category
Extenuating circumstances	Cohort 1 was scheduled to complete their final assessments starting on March 16, 2020. In addition, cohort 2 was set to have a biweekly meeting on March 16 and 18. The university study site closed campus for all activities beginning March 16, 2020.
Important modifications and mitigating strategies	
Modifications to the intervention	All in-person intervention activities were converted to videoconference delivered (Zoom). Content and structure remained the same except for hands-on cooking and recipe sampling. By summer 2020, participants were able to pick up handouts and samples of the foods on campus.
Modifications to data collection	Questionnaires were all conducted online. In-person weight and laboratory assessments were delayed until June 2020 when approval from the university was granted. The following adjustments were made to laboratory procedures to ensure social distancing and safety of participants: Appointment times were lengthened to avoid overlap between participants. For cohort 1, 24-mo weight, blood pressure, and blood samples were collected. No dual-energy x-ray absorptiometry scans or body circumference measures were taken to reduce appointment time and close contact. For cohort 2, 12-mo weight, blood pressure, blood samples, and dual-energy x-ray absorptiometry were collected. For participants who did not want to return to in-person assessments, staff provided an option for participants to have their weight assessed outside or to provide a self-reported weight. We continued the nutrition intervention components until assessments could be completed.
Impacts	COVID-19 affected delivery of in-person classes and delayed assessments. COVID-19 had important impacts on people’s nutrition, physical activity, and stress.^25,26,27,28^ This is particularly true among communities of color, which were disproportionately affected by COVID-19.^29,30^

### Statistical Analysis

Data analysis was performed from March to June 2022. The study statisticians (E.A.F. and B.H.) were blinded to group assignment when completing all analyses. Descriptive statistics were used to present baseline characteristics, and independent *t* tests were used to compare the mean number of classes attended by group. Two-sided *P *< .05 was considered statistically significant. Modified intent-to-treat analysis was conducted. Primary study aims were addressed using repeated-measures, mixed models with maximum likelihood estimation and robust computation of SEs performed using SAS statistical software version 9.4 (SAS Institute). The distributional assumption for outcomes was checked. The models included time, group, and a time-by-group interaction. The full information from the available data was used in each model to provide unbiased estimates of the intervention effect in the presence of attrition using all 4 follow-up time points. Contrasts were constructed comparing body weight (or other outcomes) at 3, 6, 12, and 24 months between groups and to estimate the changes from baseline to each time point. The model included covariates adjusting for baseline employment (employed for wages, self-employed, retired, or other), education (high school or some college, college graduate, or advanced degree), food security status (US Adult Food Security Survey Module: high or marginal vs low or very low^32^), sex, age, and time-specific medication use (for lipids, glucose, insulin, or blood pressure outcomes).

Subanalyses were also conducted to examine 12-month weight loss in cohort 1 (before COVID-19) and cohort 2 (after COVID-19 onset). The model used to compare weight change between treatment conditions was modified to examine differences in weight change between cohorts. Rather than group, time, and group-by-time interaction effects, this model contained cohort, time, and group-by-cohort effects for both treatment groups combined. Covariates were the same as in the primary weight loss model. All analyses were conducted as planned in our original analysis plan, except for the analysis by cohort, which was conducted before unblinding of the data and after analysis of the initial results to compare weight loss among participants in the study before and during the COVID-19 pandemic.

Sample size calculations have been described elsewhere.^15^ On the basis of our pilot data using both African America and White participants,^33^ we observed an SD of 4.1 kg for weight change averaged across groups. A minimum important difference of 2.3 kg (a weight difference observed in other interventions using PBDs^34^) between groups would correspond to a 0.56 effect size. To detect an effect size of 0.56 with 51 persons per group, the power is 80%. In addition, an observational study^35^ was used that examined only African American vegan participants compared with African American nonvegan participants to calculate power. In that study,^35^ African American vegan participants had significantly lower LDL cholesterol and BMI. Using the observed difference of 4.25 mg/dL (to convert to millimoles per liter, multiply by 0.0259) SD for LDL and 0.43 SD for BMI yields minimum important differences of 2.39 mg/dL for LDL and 0.24 for BMI at 80% power and a sample size of 51 participants per group. We estimated that we would have approximately 25% attrition at 12 months with a goal of recruiting a minimum of 130 participants (65 per group). See the statistical analysis plan in Supplement 1 for more information.

## Results

The CONSORT diagram for the study is shown in Supplement 1. An objective measure of body weight was considered the primary measure that represented capture of data from a participant at each time point. There were 568 participants who completed an online screening questionnaire; 409 were excluded, and 159 were randomized (77 to the vegan group and 82 to the omni group). Of the 159 participants (mean [SD] age, 48.4 [10.6] years; 126 female [79%]) who began the study, body weight was obtained for 147 (92% completion) at 3 months, 142 (89% completion) at 6 months, 121 (76% completion) at 12 months, and 100 (63% completion) at 24 months. Dietary data were obtained for 153 participants (96% completion) at 3 months, 134 (84%) at 6 months, 116 (73%) at 12 months, and 100 (63%) at 24 months. Table 3 provides the baseline demographic characteristics of participants. Dietary adherence was assessed over the 2-year study. At 3 months, 32 vegan (41%) and 20 omni (24%) participants were adherent, 23 vegan (30%) and 18 omni (22%) participants were adherent at 6 months, 27 vegan (35%) and 21 omni (26%) participants were adherent at 12 months, and 22 vegan (29%) and 20 omni (24%) participants were adherent at 24 months.

**Table 3.** Baseline Characteristics of Study Participants in the Nutritious Eating With Soul Study

Characteristic	Participants, No. (%)	
	Vegan (n = 77)	Omnivorous (n = 82)
Age, mean (SD), y	49.1 (10.5)	47.6 (10.7)
Sex		
Female	61 (79)	65 (79)
Male	16 (21)	17 (21)
Education		
High school or equivalent or some college	21 (27)	20 (24)
College graduate	29 (38)	25 (31)
Advanced degree	27 (35)	37 (45)
Occupation		
Employed for wages	54 (70)	64 (78)
Self-employed	11 (15)	8 (10)
Retired	9 (12)	5 (6)
Other^a^	3 (3)	5 (6)
Marital status		
Single	19 (24)	25 (31)
Married	39 (51)	38 (46)
Divorced or separated	13 (17)	14 (17)
Widowed	3 (4)	2 (2)
Other^b^	6 (8)	6 (8)
Body mass index, mean (SD)^c^	37.0 (6.3)	36.8 (7.5)
Food security		
Food secure	64 (83)	72 (88)
Food insecure	13 (17)	10 (12)
Energy intake, mean (SD), kcal/d	1909.0 (647.1)	2000.0 (675.6)
Body weight, mean (SD), kg	102.9 (18.9)	102.8 (23.2)
Total percentage body fat, mean (SD), total tissue percentage fat	45.2 (6.7)	44.2 (8.1)
Total lean mass, mean (SD), kg	54.4 (9.4)	54.9 (11.6)
Waist circumference, mean (SD), cm	106.5 (15.3)	107.2 (17.0)
Hip circumference, mean (SD), cm	122.5 (12.5)	122.6 (18.0)
Total cholesterol, mean (SD), mg/dL	182.1 (33.5)	173.9 (30.3)
Cholesterol-to-HDL ratio, mean (SD)	3.5 (0.9)	3.7 (0.8)
HDL cholesterol, mean (SD), mg/dL	53.8 (12.10)	48.9 (10.3)
LDL cholesterol, mean (SD), mg/dL	110.5 (30.7)	108.3 (25.2)
Triglycerides, mean (SD), mg/dL	84.2 (29.3)	83.7 (35.6)
VLDL cholesterol, mean (SD), mg/dL	16.8 (5.8)	16.7 (7.1)
Glucose, mean (SD), mg/dL	70.4 (26.0)	72.7 (27.1)
Insulin, mean (SD), μIU/L	11.6 (7.6)	13.5 (10.0)
Blood pressure, mean (SD), mm Hg		
Systolic	132.6 (17.9)	133.0 (16.5)
Diastolic	82.2 (9.9)	83.1(9.4)
Medications at baseline		
Lipids or high cholesterol	9 (12)	9 (11)
Blood pressure or hypertension	25 (33)	31 (38)
Glucose	0	0

Abbreviations: HDL, high-density lipoprotein; LDL, low-density lipoprotein; VLDL, very-low-density lipoprotein.

SI conversion factors: To convert glucose to millimoles per liter, multiply by 0.0555; HDL cholesterol to millimoles per liter, multiply by 0.0259; insulin to picomoles per liter, multiply by 6.945; LDL cholesterol to millimoles per liter, multiply by 0.0259; total cholesterol to millimoles per liter, multiply by 0.0259; triglycerides to millimoles per liter, multiply by 0.0259.

^a^ Other for employment includes being unable to work or out of work or being a student or homemaker.

^b^ Other for marital status includes being widowed or living with someone.

^c^ Body mass index is calculated as weight in kilograms divided by height in meters squared.

Table 4 provides the results of changes in the primary outcomes (body weight and lipids). There were no differences in outcomes between groups, including 12-month changes in weight (mean, –2.39 kg [95% CI, –3.48 to –1.30 kg] for the vegan group vs –2.03 kg [95% CI, –3.07 to –1.00 kg] for the omni group; *P* = .64), total cholesterol (–1.05 mg/dL [95% CI, –9.60 to 7.50 mg/dL] for the vegan group vs 1.66 mg/dL [95% CI, –7.20 to 10.50 mg/dL] for the omni group; *P* = .67), or LDL cholesterol (mean, –2.56 mg/dL [95% CI, –9.52 to 4.40 mg/dL] for the vegan group vs –0.79 mg/dL [95% CI, –7.98 to 6.40 mg/dL] for the omni group; *P* = .73). Table 5 provides changes in all secondary outcomes (kilocalories per day, body composition, glucose, insulin, and blood pressure). There were no differences between groups in any outcome (Table 4 and Table 5), or with the mean number of classes attended (30.1 classes [95% CI, 26.6 to 33.5 classes] for the vegan group vs 30.2 classes [95% CI, 26.8 to 33.5 classes] for the omni group, *P* = .99; equivalent to 59% of the classes offered) or make-up classes completed (3.3 classes [95% CI, 2.5 to 4.2 classes] for the vegan group vs 2.9 classes [95% CI, 2.1 to 3.7 classes] for the omni group; *P* = .48) over the course of the study. Mean percentage weight loss was highest at 6 months for both groups (–2.6% for vegan and –2.0% for omni). Percentage weight loss for both groups remained similar across the 12-month (–1.9% for omni and –2.2% for vegan) and 24-month (–1.9% for omni and –2.3% for vegan) assessments.

**Table 4.** Main Outcomes of Weight Loss and Changes in Lipids, by Group^a^

Outcome	Group adjusted mean (SE) [95% CI]			*P* value for difference
	Vegan (n = 77)	Omnivorous (n = 82)	Difference	
Weight change, kg				
3 mo	–2.07 (0.51) [–3.08 to –1.06]	–1.28 (0.49) [–2.25 to –0.31)	–0.79 (0.71) [–2.19 to 0.61)	.27
6 mo	–2.83 (0.52) [–3.86 to –1.80]	–2.11 (0.50) [–3.09 to –1.13]	–0.72 (0.72) [–2.13 to 0.70]	.32
12 mo^b^	–2.39 (0.55) [–3.48 to –1.30]	–2.03 (0.53) [–3.07 to –1.00]	–0.36 (0.76) [–1.86 to 1.14]	.64
24 mo	–2.46 (0.60) [–3.63 to –1.28]	–2.02 (0.56) [–3.12 to –0.93]	–0.43 (0.82) [–2.04 to 1.17]	.59
Change in total cholesterol, mg/dL	n = 73	n = 76		
6 mo	–6.64 (4.0) [–14.6 to 1.3]	0.18 (4.1) [–7.8 to 8.2]	–6.82 (5.73) [–18.1 to 4.5]	.23
12 mo^b^	–1.05 (4.40) [–9.60 to 7.50]	1.66 (4.48) [–7.20 to 10.50]	–2.71 (6.25) [–15.00 to 9.60]	.67
24 mo	1.12 (4.84) [–8.40 to 10.60]	2.50 (5.23) [–7.80 to 12.80]	–1.39 (7.11) [–15.40 to 12.60]	.85
Change in cholesterol-to-HDL ratio	n = 73	n = 76		
6 mo	–0.07 (0.08) [–0.24 to 0.09]	0.06 (0.09) [–0.10 to 0.23]	–0.14 (0.12) [–0.37 to 0.10]	.25
12 mo^b^	0.01 (0.09) [–0.17 to 0.20]	0.04 (0.09) [–0.15 to 0.22]	–0.02 (0.13) [–0.29 to 0.24]	.86
24 mo	–0.06 (0.10) [–0.26 to 0.14]	0.09 (0.11) [–0.13 to 0.31]	–0.15 (0.15) [–0.44 to 0.15]	.33
Change in HDL cholesterol, mg/dL	n = 73	n = 77		
6 mo	–0.65 (0.94) [–2.51 to 1.21]	0.20 (0.95) [–1.68 to 2.08]	–0.85 (0.1.34) [–3.50 to 1.79]	.53
12 mo^b^	–1.04 (1.03) [–3.06 to 9.99]	0.10 (1.06) [–1.99 to 2.20]	–1.14 (1.48) [–4.05 to 1.77]	.44
24 mo	1.14 (1.14) [–1.11 to 3.38]	1.63 (1.24) [–0.81 to 4.08]	–0.50 (1.68) [–3.81 to 2.82]	.77
Change in LDL cholesterol, mg/dL	n = 73	n = 76		
6 mo	–5.86 (3.26) [–12.28 to 0.56]	0.86 (3.31) [–5.65 to 7.37]	–6.73 (4.65) [–15.88 to 2.43]	.15
12 mo^b^	–2.56 (3.54) [–9.52 to 4.40]	–0.79 (3.65) [–7.98 to 6.40]	–1.77 (5.08) [–11.78 to 8.23]	.73
24 mo	0.39 (3.93) [–7.36 to 8.13]	0.62 (4.33) [–7.89 to 9.14]	–0.24 (5.84) [–11.73 to 11.25]	.97
Change in triglycerides, mg/dL	n = 73	n = 76		
6 mo	6.37 (5.75) [–4.95 to 17.69]	1.87 (5.82) [–9.59 to 13.33]	4.50 (8.19) [–11.63 to 20.62]	.58
12 mo^b^	11.02 (6.23) [–1.24 to 23.29]	10.43 (6.42) [–2.20 to 23.06]	0.60 (8.94) [–17.00 to 18.19]	.95
24 mo	6.65 (6.93) [–6.98 to 20.28]	22.02 (7.49) [7.28 to 36.75]	–15.36 (10.18) [–35.40 to 4.67]	.13
Change in VLDL cholesterol, mg/dL	n = 73	n = 76		
6 mo	1.56 (0.88) [–0.17 to 3.30]	0.33 (0.89) [–1.43 to 2.09]	1.23 (1.26) [–1.24 to 3.71]	.33
12 mo^b^	2.89 (0.96) [1.01 to 4.78]	1.38 (0.99) [–0.57 to 3.32]	1.52 (1.37) [–1.18 to 4.22]	.27
24 mo	1.49 (1.09) [–0.65 to 3.64]	0.51 (1.17) [–1.79 to 2.80]	0.99 (1.59) [–2.15 to 4.12]	.54

Abbreviations: HDL, high-density lipoprotein; LDL, low-density lipoprotein; VLDL, very-low-density lipoprotein.

SI conversion factors: To convert HDL cholesterol to millimoles per liter, multiply by 0.0259; LDL cholesterol to millimoles per liter, multiply by 0.0259; total cholesterol to millimoles per liter, multiply by 0.0259; triglycerides to millimoles per liter, multiply by 0.0259.

^a^ All models were adjusted for baseline socioeconomic status (education and employment), food security status, sex, age, and use of medications that may impact the examined outcome. For lipid outcomes, use of lipid-lowering medications at the examined time point were included in the model.

^b^ Primary outcomes occurred at the 12-month assessment measure.

**Table 5.** Changes in Secondary Outcomes of Energy Intake, Body Composition, and Glucose, Insulin, and Blood Pressure, by Group^a^

Outcome	Group adjusted mean (SE) [95% CI]			*P* value for difference
	Vegan (n = 77)	Omnivorous (n = 82)	Difference	
Change in energy intake, kcal/d				
3 mo	–624.1 (72.1) [–765.8 to –482.4]	–578.9 (69.2) [–714.9 to –442.9]	–45.2 (99.9) [–241.6 to 151.3]	.65
6 mo	–485.2 (76.3) [635.1 to 335.3]	–504.1 (72.0) [–645.6 to –362.6]	18.9 (104.9) [–187.2 to 225.1]	.86
12 mo^b^	–440.2 (79.5) [–596.3 to –284.1]	–534.0 (75.8) [–682.9 to –385.1]	93.8 (109.8) [–121.9 to 309.4]	.39
24 mo	–555.4 (83.8) [–720.1 to –390.7]	–461.0 (79.5) [–617.1 to –304.9]	–94.4 (115.5) [–321.2 to 132.4]	.41
Change in total percentage body fat, total tissue percentage fat				
12 mo^b^	–0.32 (0.30) [–0.91 to 0.27]	–0.29 (0.29) [–0.87 to 0.29]	–0.03 (0.42) [–0.86 to 0.80]	.94
Change in total lean mass, kg				
12 mo^b^	–1.51 (0.30) [–2.11 to –0.90]	–1.26 (0.30) [–1.85 to –0.66]	–0.25 (0.43) [–1.10 to 0.60]	.60
Change in waist circumference, cm^c^				
6 mo	1.00 (0.79) [–0.55 to 2.57]	0.06 (0.75) [–1.42 to 1.53]	0.95 (1.09) [–1.19 to 3.10]	.38
Change in hip circumference, cm^c^				
6 mo	–0.60 (0.79) [–2.16 to 0.97]	–0.39 (0.75) [–1.87 to 1.09]	–0.20 (1.09) [–2.36 to 1.95]	.85
Change in glucose, mg/dL	n = 73	n = 77		
6 mo	3.59 (2.61) [–1.54 to 8.72]	1.63 (2.62) [–3.53 to 6.78]	1.96 (3.69) [–5.31 to 9.23]	.60
12 mo^b^	–0.96 (2.83) [–6.54 to 4.61]	1.75 (2.92) [–4.00 to 7.49]	–2.71 (4.06) [–10.71 to 5.29]	.51
24 mo	4.09 (3.15) [–2.11 to 10.29]	2.34 (3.08) [–3.73 to 8.41]	1.75 (4.40) [–6.91 to 10.41]	.69
Change in insulin, μIU/L	n = 73	n = 76		
6 mo	–1.19 (1.05) [–3.25 to 0.87]	–2.10 (1.06) [–4.18 to –0.02]	0.91 (1.49) [–2.02 to 3.83]	.54
12 mo^b^	–0.88 (1.13) [–3.11 to 1.36]	–2.69 (1.17) [–4.99 to –0.38]	1.81 (1.63) [–1.40 to 5.02]	.27
24 mo	–1.74 (1.26) [–4.23 to 0.74]	–1.63 (1.37) [–4.32 to 1.06]	–0.12 (1.86) [–3.77 to 3.53]	.95
Change in systolic blood pressure, mm Hg				
6 mo	0.41 (1.53) [–2.60 to 3.42]	–0.74 (1.46) [–3.61 to 2.13]	1.15 (2.12) [–3.01 to 5.31]	.59
12 mo^b^	–2.25 (1.62) [–5.44 to 0.93]	–3.16 (1.55) [–6.22 to –0.10]	0.91 (2.25) [–3.51 to 5.32]	.69
24 mo	0.25 (1.75) [–3.18 to 3.68]	–0.43 (1.65) [–3.68 to 2.82]	0.68 (2.40) [–4.05 to 5.40]	.78
Change in diastolic blood pressure, mm Hg				
6 mo	–0.80 (0.87) [–2.50 to 0.91]	–0.30 (0.83) [–1.93 to 1.33]	–0.50 (1.20) [–2.86 to 1.86]	.68
12 mo^b^	–1.27 (0.92) [–3.08 to 0.54]	–2.05 (0.88) [–3.78 to –0.32]	0.78 (1.27) [–1.72 to 3.29]	.54
24 mo	–0.07 (0.99) [–2.02 to 1.88]	–0.53 (0.94) [–2.37 to 1.32]	0.46 (1.36) [–2.22 to 3.14]	.74

SI conversion factors: To convert glucose to millimoles per liter, multiply by 0.0555; high-density lipoprotein cholesterol to millimoles per liter, multiply by 0.0259; insulin to picomoles per liter, multiply by 6.945.

^a^ All models were adjusted for baseline socioeconomic status (education and employment), food security status, sex, age, and use of medications that may impact the examined outcome. For glucose and insulin outcomes, use of diabetes-related medications at the examined time point were included in the model. For blood pressure, use of hypertensive medications at the examined time point were included in the model.

^b^ Primary outcomes occurred at the 12-month assessment measure.

^c^ Because of the COVID-19 pandemic, body circumference measures were not taken after the 6-month assessments (which were all conducted prior to the pandemic).

Weight loss that occurred in cohort 1 (67 participants) was compared with weight loss that occurred in cohort 2 (92 participants). Weight loss did not differ at 3 months (mean, cohort 1 vs cohort 2, –1.65 kg [95% CI, –2.76 to –0.53 kg] vs –1.56 kg [95% CI, –2.50 to –0.62 kg]; *P* = .90) or 6 months (mean, cohort 1 vs cohort 2, –2.65 kg [95% CI, –3.79 to –1.52 kg] vs –2.26 kg [95% CI, –3.22 to –1.31 kg]; *P* = .60), which both occurred before the COVID-19 pandemic. There were differences at both subsequent time points (12-month outcomes were assessed before COVID-19 for cohort 1 and after onset of the pandemic for cohort 2). Cohort 1 lost more weight than cohort 2 (mean, –3.45 kg [95% CI, –4.67 to –2.22 kg] vs –1.24 kg [95% CI, –2.24 to –0.25 kg]; *P* = .01) at month 12 and at month 24 (mean, –3.26 kg [95% CI, –2.66 to –0.65 kg] vs –1.65 kg [95% CI, –4.54 to –1.98 kg]; *P* = .05).

## Discussion

NEW Soul is one of the first long-term, randomized clinical trials comparing the effects of 2 different healthy soul food diets on changes in body weight and CVD risk factors among African American individuals. Although most outcomes were in the hypothesized direction, there were no differences between the groups, and the magnitude of changes overall was small. It is possible that the COVID-19 pandemic impacted the results. Weight loss was greatest at 6 months, which is similar to other weight loss interventions among adults with^36^ and without^37^ type 2 diabetes, and was also the last weight measurement taken before COVID-19.

It is also possible that the diets selected were too similar to produce significant differences. Both diets were based on the Oldways African Heritage Diet, which is heavily focused on PBDs. The 6 lessons in the A Taste of African Heritage program all centered around plants (herbs and spices, greens, whole grains, beans and rice, tubers and stews, and fruits and vegetables).^22^ Although the vegan diet recommended that participants abstain from meat, the omni diet group was told to follow a meat-reduced diet (<5 oz [140 g] of meat per day). In the end, energy intake and adherence decreased similarly between groups and across time points. Prior research has shown that people following a vegan diet have lower energy intakes and higher fiber intakes (both of which should promote weight loss^38^) than those following an omnivorous diet.^39^

In addition, the COVID-19 pandemic had significant impacts on diet and physical activity interventions,^40^ and the NEW Soul study was no exception. Cohort 2 was impacted by the COVID-19 pandemic to a greater degree, with their in-person classes moving to remote before their 1-year assessment. It is possible the pandemic diluted the intervention effect, making it difficult to detect differences between the groups at 12 and 24 months. Other studies^41,42^ have found that the COVID-19 lockdown orders had impacts on weight and eating behaviors. Studies showed that weight gain was common among adults in the US during the pandemic. During the COVID-19 lockdown, people also reported high levels of stress and stress-related eating.^41,43,44^ Few studies have examined the impact of COVID-19 pandemic on African American individuals’ body weight, making this one of the few studies to be conducted in this area.

Few weight-loss and CVD risk factor reduction randomized clinical trials have been conducted solely among African American individuals. Many previous weight loss interventions have included both African American and White adults, included mostly women,^45,46,47^ and had limited representation of African American men.^48^ These interventions have consistently found greater weight loss among White women compared with African American women.^45,46,47,49^ For example, a systematic review^45^ found that African American women lost less weight than their White counterparts, with weight changes among African American women ranging from a gain of 0.5 kg to a loss of 4.7 kg. In a weight loss intervention among African American breast cancer survivors, a 12-month weight loss program led to a mean weight loss of 2.7 kg.^50^ These ranges are similar to what was observed in the present study. Differences in weight loss between African American and White individuals have been attributed to potential differences in energy expenditure (through physical activity),^51^ having lower energy requirements,^47^ or having lower engagement with the intervention content.^46^

There have also been limited studies conducted with African American individuals that examine the adoption of different dietary patterns. As part of the Heart Healthy Lenoir Project, African American individuals who enrolled in a 6-month Mediterranean diet intervention to reduce CVD risk factors had improvements in dietary intake, physical activity, and blood pressure, but saw modest weight loss.^52^ The Dietary Approaches to Stop Hypertension diet (similar to the omni group in the present trial) has produced significant reductions in blood pressure among African American individuals.^53^

### Limitations

The results of this study should be considered within the context of its limitations. The study population was highly educated, mostly women, and living in the South and, therefore, may not be generalizable to other populations. Although objective measures were used for weight, body composition, and CVD risk factors, dietary intake and food security status were self-reported. High-density lipoprotein cholesterol levels can differ by sex,^54^ and results for lipids were not stratified by sex, limiting some of the interpretation. Although assessors for all outcomes and the study statistician were blinded to group assignment, it was not possible to blind the study interventionist. Participant groups met at different times, but it is possible that participants shared information about their diet with the other group. Furthermore, the COVID-19 pandemic impacted the ability to deliver the intervention with the same modality for the 2 cohorts and may have impacted attrition, with 37% of participants not completing 24-month assessments.

## Conclusions

In this randomized clinical trial examining weight loss and CVD risk factor reduction among African American individuals, there were no significant differences in weight loss and changes in lipids and blood pressure among African American individuals randomized to either healthy soul food vegan or omnivorous diets. The changes in intervention delivery that necessitated a switch to online classes most likely reduced the impact of the study, lessening the differences between the groups. Future research should examine additional strategies to enhance adherence to PBDs, such as testing the intervention in a nonuniversity, community-based setting or providing ready-to-eat meals.

## Notes

Supplement 1.
Trial Protocol and Statistical Analysis Plan

Supplement 2.
Data Sharing Statement
