# Supplementary material for: Effect of a Plant-Based vs Omnivorous Soul Food Diet on Weight and Lipid Levels Among African American Adults: A Randomized Clinical Trial
Source: JAMA Netw Open. 2023 Jan 12;6(1):e2250626. doi: 10.1001/jamanetworkopen.2022.50626 (PMC9857469; doi:10.1001/jamanetworkopen.2022.50626)
Supplement: Supplement 1. — Trial Protocol and Statistical Analysis Plan [file jamanetwopen-e2250626-s001.pdf]

Nutritious Eating with Soul Protocol Documents

1. NEW Soul Protocol Overview: Pages 2-29.
2. COVID measurement adjustments: Pages 30-32.
3. NEW Soul Manual of operations: 33-91.

Research Protocol: The Nutritious Eating with Soul (NEW Soul) Study

Revision date 10-19-2019

1. Study rationale

In the US, African Americans (AAs) have the highest rates of obesity and heart disease as compared with whites and Hispanics [1]. More AA men and women die from cardiovascular disease (CVD) than any other chronic disease condition [2]. Although CVD mortality has declined over the previous four decades, rates of CVD mortality among AAs have remained disproportionately high [3]. Overweight and obesity are associated with a number of chronic diseases, including type 2 diabetes, hypertension and CVD [4, 5], as well as an increased risk of several cancers [6, 7].

Vegan diets "are healthful, nutritionally adequate, and may provide health benefits in the prevention and treatment of certain diseases" (Position of the Academy of Nutrition and Dietetics) [8]. Data from the Adventist Health Study-2 (AHS-2) have shown that people following vegan diets have the lowest body mass indices (BMIs) and the lowest prevalence of type 2 diabetes compared to other diet patterns [9]. In addition, findings from the European Prospective Investigation into Cancer and Nutrition (EPIC-Oxford) study have shown that vegans gain significantly less weight as they age compared with omnivores. Converting to a more plant-based diet also appears to be protective against weight gain, as does following a pesco-veg diet in women [10].

Research from the AHS-2 has found that compared with AA omnivores, AA vegetarians/vegans had significantly lower risk of hypertension, diabetes, and high total and LDL cholesterol [11]. In addition, vegan diets were protective against overall cancer incidence and female-specific cancers as compared with four other plant-based dietary patterns [12]. Plant-based diets may confer even more protection against chronic disease for AAs than for whites. For example, in an AHS-2 cohort study examining diabetes risk, diet pattern, and race, researchers found that AAs following vegan, vegetarian, or semi-vegetarian diets had a lower risk of diabetes than those following an omnivorous diet [13].

The objective of the Nutritious Eating with Soul (NEW Soul) study is to determine which diet allows for greater sustained changes in body weight, lipids, and blood pressure.

2. Study process

Adults with obesity or overweight (BMI 25-49.9 kg/m^2) who self-identified as AA will be randomized to follow one of two diets emphasizing soul food cuisine: (1) Vegan: whole foods, plant-based vegan diet or (2) Omni: low-fat omnivorous diet. This randomized behavioral intervention has a recruitment goal of a minimum of 130 participants over two cohorts separated by one year.

2.1 Screening

Participants are to be recruited through media interviews (TV, radio, and newspaper), radio commercials, community outreach, and word of mouth. Participants will be directed to a study website where they can learn more about the study and complete an online screener questionnaire. Participants complete the Physical Activity Readiness Questionnaire [14] as part of the online screener and are asked to get physician consent to participate if they are currently on medications for blood pressure or a heart condition. If they indicate "yes" on any of the other questions (e.g., "Do you feel pain in your chest when you perform physical activity?"), then they are excluded from the study. Participants who are not eligible are sent an email thanking them for their interest in the study. Participants who are eligible are contacted by a study coordinator to complete a brief phone screener. Eligible and interested participants are given a choice of meeting times to attend an orientation session. The nine inclusion and six exclusion criteria are presented in Table 1. Because of the level of details necessary to explain the study, the orientation session is split into two separate sessions. Participants receive training on how to complete the 24-hour recall and complete one at the orientation in order to have time to ask questions and receive assistance from study staff. In addition, participants are provided with an overview of what

the study entails. This includes a detailed description of each diet, along with sample meal ideas. Because vegan diets are unfamiliar to most participants, dinner is provided to participants that is catered from a local vegan soul food restaurant. This allows participants to get a better understanding of the diets in the study. Finally, participants will sign up for their baseline laboratory assessment time.

2.2 Baseline data collection

Data are collected both in-person and online (Table 2). Data collected includes survey-based data, dietary recalls, anthropometric data, and laboratory assessments. More information about these assessments is provided below.

2.3 Randomization

Participants who complete all baseline assessments are randomized to one of the two diet conditions. Randomization is stratified by gender and carried out in blocks of 10 to ensure equal distribution between the groups (since randomization is conducted on a rolling basis as participants complete assessments). The study statistician creates the allocation sequences with a computerized random number generator and a research assistant who is blinded to participant identification randomizes the participants into each group using the sequences.

2.4 Single-blind

The NEW Soul study is single-blinded, as it is not possible to blind study participants to their study assignment. It is also not possible to blind intervention staff to study assignment. All assessment staff are blinded to group assignment, however, and staff involved in randomization are blinded to study participant identity. Participants are instructed not to tell assessment staff of their assignment.

2.5 Staggered cohorts

To accommodate a minimum of 130 participants in the study, the intervention is conducted in two cohorts separated by one year. Cohort 1 began the study in May 2018 and Cohort 2 began the intervention in June 2019. Cohorts are utilized and staggered in order to ensure adequate recruitment, accommodate space limitations of our demonstration kitchen and teaching classroom, guarantee appointments for laboratory assessments, and account for the number of available accelerometers.

2.6 Hypotheses

The NEW Soul study has one primary aim and one secondary aim, along with corresponding hypotheses. The primary aim is to examine differences in risk factors at 12 months for CVD (changes in lipids, glucose, insulin, and blood pressure) and body weight between participants randomized to the omni or vegan group. We hypothesize that CVD risk factors and body weight will improve to a greater extent in the vegan group as compared with the omni group. The secondary aim is to examine long-term changes in CVD risk factors at body weight at 24 months. We hypothesize that the greatest improvements in CVD risk factors and body weight will be seen within the vegan group as compared with the omni group, demonstrating maintenance of health-related behaviors.

3. Intervention Protocol

3.1 Group-based classes

The NEW Soul intervention consists of weekly in-person meetings lasting 75 minutes for six months (26 meetings), followed by bi-weekly meetings for six months (13 meetings), and then monthly meetings for the last 12 months (12 meetings). Participants are given a choice of two different meeting times on two different days in order to accommodate work, childcare, and church schedules. All classes for both cohorts and both groups are led by the same core staff (MS, RDN-trained nutrition interventionist, MPH-trained project manager, and a discussion facilitator from the community). In addition, AA soul food

restaurant owners and chefs provide cooking demonstrations for study participants or provide food items to sample.

Treatment fidelity is assessed on three occasions per year by a research assistant in class, without knowledge of the intervention staff. A checklist of required class elements is used to ensure A) that required topics are covered and B) both classes are receiving equal treatment in terms of topics covered and duration of the classes. These treatment fidelity reports are then reviewed during the weekly study staff team meetings and any issues are addressed in future classes. Participants are given a choice of two days and class times per week to attend their intervention group sessions. While participants could attend either day and time for their group (and switch between those days and times each week), they are not permitted to attend a day and time that is not for their assigned diet.

3.2 Remotely delivered content

In addition to the face-to-face group classes, content is provided remotely. Each week when a face-to-face class is offered, participants also receive a weekly email. This email provides a link to the study participant website where participants can access the handouts from the class, copies of the recipes, and additional notes from study staff (such as feedback from participants about how to alter a recipe). If a participant misses a class, they have the option of completing a make-up class online. A video of the class slides with instruction about what is covered in class will be made available to participants and they can complete a brief quiz at the end of the make-up session to verify completion. In addition to these online make-up sessions, monthly face-to-face sessions are offered during lunch time for participants who wish to make up content missed in person.

At six months, when the frequency of the weekly meetings changes to bi-weekly, participants are provided with private Facebook groups for their diet groups. This allows participants to continue to provide social support to one another and allows study staff to post information about their diets on

weeks when classes are not held. At 12 months, when classes change frequency to monthly meetings, participants receive a podcast, newsletter, or face-to-face meeting each week. The schedule of delivery is to provide a podcast on the first week of the month, followed by an online newsletter in the second week, a face-to-face meeting in the third week, and another podcast in the fourth week of the month. This allows for weekly delivery of content for the study.

Podcasts are developed using Social Cognitive Theory [15] and are designed to emphasize behavioral strategies useful for dietary maintenance. Specific diets are not mentioned, and the podcasts are the same for both groups. Weekly newsletters provide information about upcoming classes and recipes and are different for each of the diet groups.

3.3 Intervention diets

The objective of the NEW Soul study is to compare two different diets for CVD prevention. Participants are randomized to follow either a vegan or omnivorous (omni) diet. Both diets focus on soul food and traditional African cuisine, and both emphasize plant-rich, low-fat eating styles (Table 3). The vegan diet recommends a whole food, plant-based dietary approach [16, 17], meaning a focus on minimally processed plant foods and avoiding refined foods, including oils. Participants are encouraged to meet their fat requirements through whole foods (e.g., nuts, nut butters, avocados, and seeds). The omni diet follows the Therapeutic Lifestyle Changes dietary guidelines in order to have guidance on portion sizes for foods like lean meats [18]. Both diets are guided by the Oldways African Heritage Pyramid [19], which emphasizes intake of fruits, vegetables, particularly leafy greens and tubers, and whole grains. The pyramid was adapted for the vegan diet group (e.g., legumes in place of meat). The omni diet also included the intake of fish, poultry, and low-fat dairy, and modest amounts of red meat, as outlined by the Oldways pyramid.

3.4 Theory-based content and similarities in intervention content and strategies

The dietary recommendations are the only differences between the groups. Both groups have the same intensity with regards to intervention delivery and have the same instructors and discussion facilitators. Social Cognitive Theory [15] guided the class curriculum with a specific emphasis on building self-efficacy and goal setting. Participants follow a gradual approach to adopting their diets in order to build self-efficacy for dietary change. They focus only on following their assigned diet for breakfast the first week, then add in lunch in week two, and finally add dinner for week three. Participants are provided with training on how to write a SMART goal [20] and then are asked to create a SMART goal each week around the topic discussed. Goals from the previous week are then discussed at the beginning of each class. The general class structure for the NEW Soul study and the constructs from Social Cognitive Theory that are targeted in each class are detailed in Table 4.

In addition to dietary goals, both groups are given the same exercise and stress management goals and techniques. While the focus of the NEW Soul study is to examine the impact of diet on health outcomes, physical activity and stress management were also components of the classes. These health behaviors were included because participants were interested in improving in these areas and these behaviors are also important components of cardiovascular health and weight loss. It is important to note, however, that the recommendations for these behaviors were identical between the groups. For physical activity, participants are encouraged to meet the Physical Activity Guidelines for Americans including achieving 150-300 minutes of moderate-intensity aerobic activity [21]. Participants are encouraged to build up to that goal and achieve it through activities they enjoy or can do for free (e.g., brisk walking). In addition, participants are encouraged to do strength-training activities at least twice per week. Participants are given resistance bands and shown how to use them for strength-training activities. Further, participants are encouraged to find ways to regularly stretch to increase flexibility. A yoga instructor attends two of the classes to demonstrate chair yoga techniques, and a massage therapist attends a class session to demonstrate stretching techniques. The yoga instruction is also used to demonstrate stress

management techniques. Participants are given stress management information based on the Diabetes Prevention Program [22], and a licensed counselor discusses ways to handle emotional and stress eating.

3.5 Addressing dietary adherence with study participants

Participants in Cohort 1 are emailed a link to a brief survey each week that a face-to-face class is held. This survey asks participants about their degree of adherence to dietary guidelines for their group. The questionnaire has 10 food categories (e.g., whole grains, legumes, red meat, eggs, etc.) and asks participants to select the frequency in which they consumed those food groups over the last seven days. Participants can also indicate if they would like to be contacted regarding their diet adherence. Due to low completion rates for the online survey, a brief, paper, in-class survey was provided to Cohort 2 participants (with the option to complete online if a participant missed a class). Participants having difficulty adhering to the dietary recommendations (and indicate they are willing to be contacted) are contacted via phone by a study coordinator. Adherence calls to participants who are willing to be contacted are conducted weekly during the first six months, bi-weekly for the next six months, and monthly for the last 12 months of the study. The study coordinator asks the study participant to identify barriers to following the diet and then collaboratively discusses with the participant potential solutions (e.g., batch cooking to save time) and resources (e.g., recipes) that may be helpful. If a participant continues to have difficulties, they are offered in-person counseling with a registered dietitian. These additional contacts are offered to all participants in order to ensure participants have the ability to ask questions and problem solve outside of class time if questions were not adequately address during classes.

3.6 Methods of contacting participants

To accommodate the varied ways that participants want to be contacted, a study database includes methods of emailing or texting participants. Participants are sent information about upcoming classes, reminders about assessment appointments, and reminders to complete surveys via text message, email, and phone. The study database tracks contact methods and frequency.

3.7 Iterative intervention adaptation and development (updated changes after cohort 1)

Based on feedback from Cohort 1, the order of the first classes were slightly altered for Cohort 2 (Table 5). Namely, the grocery store tour, which occurred in week 7 for Cohort 1 was moved up to Week 4 for Cohort 2. In addition, participants in Cohort 1 requested that cooking demonstrations and recipes for more typical and traditional soul foods occur earlier in the intervention versus a focus on recipes from the Oldways African Heritage program. Therefore, sample breakfast, lunch, and dinner meals were prepared in the initial weeks of the intervention for Cohort 2 that more closely mirrored traditional soul food, such as vegan or low-fat versions of grits, macaroni and cheese, and red beans and rice. In addition, Cohort 1 requested more frequent hands-on cooking classes and more guest chefs from the community. Both were added to Cohort 1 and were continued for Cohort 2. While these changes occurred in Cohort 2, it is important to note that they occur for both diet groups in Cohort 2. Because these changes were fairly minor, did not include major changes in the intervention design or content, and occurred for both diet groups equally, we do not anticipate an impact on study outcomes. An overview of class topics for the study can be found in Table 5.

4. Assessment protocol

All assessment measures are collected at baseline, six months, 12 months and 24 months except for DXA scans, which are not collected at 6 months (Table 2). Assessments take place at the Clinical Exercise Research Center at a large research university. In addition, surveys and dietary recalls are also collected

at three months. Below details how primary outcomes (weight and CVD risk factors) and secondary outcomes (e.g., diet, body fat, etc.) were assessed.

4.1 Dietary assessment

Dietary intake is assessed using the National Cancer Institute's Automated Self-administered 24-hour recall (ASA24) [23]. Intake is assessed on three unannounced days including one weekend day (Friday, Saturday, or Sunday) and two weekdays at each assessment time point. Dietary recalls are collected within a three-week window to allow for enough time to contact participants and allow them to complete all three recalls. The ASA24 can be accessed either online or via a smartphone or tablet. Participants with low computer literacy or without consistent access to these devices are offered the ability to complete the recalls over the phone with an interviewer who used the ASA24 system to guide participants through the recall or can be completed in person in the university computer lab. As described earlier, all participants receive an in-person training on ASA24 at baseline and complete their initial dietary recall in-person in order to have opportunities to ask questions. After the initial recall, all other recalls are completed remotely. Participants are contacted on unannounced days to complete their recall. The ASA24 system allows for the participant to complete the recall at any time on the day in which they are prompted up until midnight. If a recall is not completed on that day, participants are then prompted on another non-consecutive unannounced day. Participants are contacted via text message, email, and phone to remind them to complete a recall.

4.2 Physical activity assessment

Physical activity is assessed via accelerometers (GT1M model) worn on the hip. Participants are instructed on how to wear the accelerometers at their orientation session. Participants are asked to wear the monitors during all activities except sleeping and those that involved water (bathing, swimming, etc.) and to wear them for 10 days. The goal is to have a minimum of 4 days with at least 10

hours of wear per day, including at least one weekend day. Accelerometers are checked upon return and those without the minimum required data are asked to wear the monitors again. Actigraph data are converted from counts based on 60-second epochs to categories of inactivity or physical activity using cutpoints from Freedson [24]. Activity counts are classed as sedentary (<1.5 METs;<=100 cpm), light (1.5 to 2.9 METS; 100–1951cpm), moderate (3.0–5.9 METS; 1952–5724cpm), or vigorous (≥6 METS; ≥5725cpm). Any continuous sequence of 60 or more zero counts is considered a period of Actigraph non-wear. For each wave of data collection (baseline through 24-month) each participant's activity is summarized into average minutes per day of inactivity and physical activity categories of light, moderate, vigorous, and combined moderate-to-vigorous physical activity. Only days on which wear time was complaint with the 10-hour minimum are used in the activity summary variables.

In addition to the accelerometers, participants complete the International Physical Activity Questionnaire - Short Form (IPAQ) [25]. The IPAQ provides an additional way to collect data on physical activity and provided information on total physical activity in metabolic equivalent for task (MET)-minutes/week and time spent sitting.

4.3 Weight, height and waist circumference

Height is measured at baseline only using a calibrated, wall-mounted stadiometer (Model S100, Ayerton Corp., Prior lake, MN). A calibrated digital scale (Healthometer® model 500 KL, McCook, IL) is used to collect body weight at each assessment time point. A spring-loaded tape measure is used for all waist circumference measurements. Waist circumference is measured at the iliac crest [26], and hip circumference is measured at the maximum protuberance of the buttocks [27]. Two measurements are taken and if they are not within 4 mm of one another, then a third measurement is taken.

4.4 Dual-energy X-ray absorptiometry (DXA) scan

Upon arrival at the Clinical Exercise Research Center, participants who are not wearing appropriate clothing (such as sweatpants and a t-shirt), are asked to change into hospital “scrubs.” A DXA scan is conducted to assess lean tissue mass and fat mass for the entire body, as well as for the trunk only. Intra-instrument variability for DXA is <5.2%, and DXA has been shown to be valid across differences in race, gender, body fatness and size and athletic status [28]. A certified technician performs and provides the results of each DXA scan to study personnel. The body composition assessment takes approximately 45 minutes. Fat (g) and lean (g) mass of arms, legs, trunk, and total body is recorded.

4.5 Blood pressure

After a 5-minute rest period, blood pressure is assessed using an Omron Hem 705 CP Auto Inflate Blood Pressure Monitor. A minimum of two readings (and a maximum of four) are taken, and the average of those readings is used. If there is a >5 mmHg difference between the first and second readings, an additional one or two readings are obtained, and the average of these multiple readings is used [29].

4.6 Blood samples and analysis

A lipid panel (total, LDL and HDL cholesterol, and triglycerides) and fasting glucose and insulin are performed at baseline, six months, 12 months, and 24 months. These tests are performed on serum or plasma using commercially available assay kits. All samples from the same participant will be analyzed on the same plate and, thus, under the same conditions. Therefore, participants do not receive any of the results of these tests until after all cohorts have completed their 24-month assessments. A standard curve will be constructed using standards provided in the kits, and the sample concentrations will be determined from the standard curves. Inter-assay and intra-assay variability will be assessed.

4.7 Psychosocial questionnaires

Participants complete a variety of questionnaires at baseline, three months, six months, 12 months, and 24 months (Table 2). The following are assessed as part of the questionnaires: demographic variables

(collected at baseline only), socioeconomic status variables (education, income, own or rent home, etc.), use of nutrition assistance programs, composition of family household, and environmental changes [30, 31] (changes in neighborhood, housing, access to food, etc.), self-efficacy (SE) for adoption of healthy diets [32], medications (current medications and dosages and any changes), dietary acceptability [33-35], perceived stress [36], perception of food spending [37], health-related quality of life [38], food insecurity [39], physical activity [25], dietary restraint, disinhibition, and hunger [40], appetite for palatable foods [41].

4.8 Data safety and monitoring

NIH requires a Data Safety and Monitoring plan (DSMP) and a board (DSMB) consisting of three NIH-funded researchers from the same institution who are not involved with the study. The DSMB receives a report semi-annually from study staff that details subject accrual, status of enrolled subjects, adherence data regarding intervention attendance, and any adverse events. Any serious adverse events are to be reported immediately to the institution's IRB and DSMB. The role of the DSMB is to review the data and results of any interim analyses from an ethical standpoint, to ensure the safety, rights and wellbeing of the trial participants. In addition to reports provided via email, the DSMB also meets in-person on an annual basis and the chair of the DSMB prepares a confidential report advising the PI on whether the trial should continue or not.

4.9 Process evaluation

Process evaluation allows for the examination of a program's reach and implementation fidelity and participants' self-reported impressions, satisfaction, and behavior [42]. Process data are collected before, during, and after the intervention. The process evaluation plan is based on Saunders, et. al.[43] and includes assessment of reach, attendance and participation, dose, treatment fidelity, and compatibility/satisfaction. An online database was created that allows study team members to track

attendance and utilization of intervention materials, as well as contacts with participants (i.e., appointment reminders) and completion of make-up classes.

5. Statistical Analysis Plan

5.1 Sample size justification

The primary aims for the NEW Soul study are, using a randomized design, determine the impact at 12 months of two different, culturally tailored diets (omni and vegan) on changes in (Primary Aim 1) risk factors for CVD, including LDL cholesterol and blood pressure, and (Primary aim 2): body weight. The NEW Soul study is powered to detect differences in both CVD risk measures and weight loss. Weight loss has been used to determine power in other NIH-funded dietary trials in which both CVD risk factors and body weight were outcomes [44, 45]. Based on our pilot data using both AA and white participants, we observed a standard deviation (SD) of 4.1 kg for weight change averaged across the arms. A minimum important difference of 2.3 kg between arms would correspond to a 0.56 effect size. A 2.3 kg weight difference is similar to what has been observed in other weight loss interventions using plant-based diets [46]. Based on SDs from previous intervention studies using both AA and white participants [47, 48], for changes in waist circumference, diastolic blood pressure, and fasting glucose, this effect size corresponds to minimum important differences of, respectively, 0.50 cm, 4.8 mmHg, and 28 mg/dL. To detect an effect size of 0.56 with 51 persons per arm, the power is 80%. In addition, we also used observational studies that examined only AA vegan participants as compared with AA non-vegan participants to calculate power. In one such study [49], AA vegan participants had significantly lower LDL cholesterol and BMI as compared to non-vegan AA participants. Using the observed 0.11 mmol/L SD for LDL and 0.43 kg/m^2 SD for BMI yielded minimum important differences of 0.062 mmol/L for LDL and

0.24 for BMI at 80% power and a sample size of 51 participants per group. We accounted for ~25% attrition (by month 12) with a goal of recruiting a minimum of 130 participants (65 per group).

5.2 Statistical analyses

Data from all study measures will undergo initial data cleaning to identify potential outliers, assess normality and enumerate loss to follow-up and other missing data. Adherence will be examined, at a descriptive level, separately for men and women. Sex may be associated with adherence or primary outcomes; therefore, randomization was balanced with respect to sex in addition to its inclusion as a covariate in outcome models. Primary study aims will be addressed using repeated-measures, mixed models with maximum likelihood estimation and robust computation of standard errors as provided by PROC MIXED in the SAS® system. Under the assumption that missing data are missing at random, these models provide an intent-to-treat analysis in the presence of attrition after baseline. For each component of CVD risk and weight, a model will be estimated using factors of time (baseline vs. 12 months), intervention-arm (omni vs. vegan) and time by intervention-arm interaction. The model will include covariates adjusting for any differences due to socioeconomic status, BMI, use of nutrition assistance programs, composition of family household, self-efficacy for eating behaviors, sex, age, and change in medication or physical activity. A measure of diet adherence (assessed by absence of proscribed foods on dietary recalls as has been used in previous studies [50, 51]) will also be used as a covariate in each model. In addition to the models for the primary outcome (changes in body weight, lipids, glucose, insulin, and blood pressure), a continuous measure of compliance will be modeled using the same time, intervention-arm, interaction term, and covariates. The same models for CVD risk factors and weight will be used to address our secondary aim examining maintenance at 24 months, as well as in assessing other continuous outcomes, including changes in psychosocial variables, body composition and markers of inflammation.

References

[1] D. Lloyd-Jones, R.J. Adams, T.M. Brown, M. Carnethon, S. Dai, G. De Simone, T.B. Ferguson, E. Ford, K. Furie, C. Gillespie, A. Go, K. Greenlund, N. Haase, S. Hailpern, P.M. Ho, V. Howard, B. Kissela, S. Kittner, D. Lackland, L. Lisabeth, A. Marelli, M.M. McDermott, J. Meigs, D. Mozaffarian, M. Mussolino, G. Nichol, V.L. Roger, W. Rosamond, R. Sacco, P. Sorlie, R. Stafford, T. Thom, S. Wasserthiel-Smoller, N.D. Wong, J. Wylie-Rosett, Heart Disease and Stroke Statistics—2010 Update: A Report From the American Heart Association, Circulation 121(7) (2010) e46-e215.
[2] American Heart Association. African Americans & Cardiovascular Diseases. https://www.heart.org/idc/groups/heart-public/@wcm/@sop/@smd/documents/downloadable/ucm_319568.pdf. Accessed November 12, 2015., (2013).
[3] G.A. Mensah, Black and Minority Health 2019, Journal of the American College of Cardiology 74(9) (2019) 1264.
[4] A.H. Mokdad, E.S. Ford, B.A. Bowman, W.H. Dietz, F. Vinicor, V.S. Bales, J.S. Marks, Prevalence of obesity, diabetes, and obesity-related health risk factors, 2001, Jama 289(1) (2003) 76-9.
[5] A. Must, J. Spadano, E.H. Coakley, A.E. Field, G. Colditz, W.H. Dietz, The disease burden associated with overweight and obesity, Jama 282(16) (1999) 1523-9.
[6] E.E. Calle, C. Rodriguez, K. Walker-Thurmond, M.J. Thun, Overweight, obesity, and mortality from cancer in a prospectively studied cohort of U.S. adults, N Engl J Med 348(17) (2003) 1625-38.
[7] T. Pischon, U. Nothlings, H. Boeing, Obesity and cancer, The Proceedings of the Nutrition Society 67(2) (2008) 128-45.
[8] W.J. Craig, A.R. Mangels, Position of the American Dietetic Association: vegetarian diets, Journal of the American Dietetic Association 109(7) (2009) 1266-82.
[9] S. Tonstad, K. Stewart, K. Oda, M. Batech, R.P. Herring, G.E. Fraser, Vegetarian diets and incidence of diabetes in the Adventist Health Study-2, Nutr Metab Cardiovas (2011) 1-8.
[10] M. Rosell, P. Appleby, E. Spencer, T. Key, Weight gain over 5 years in 21,966 meat-eating, fish-eating, vegetarian, and vegan men and women in EPIC-Oxford, Int J Obes (Lond) 30(9) (2006) 1389-96.
[11] G. Fraser, S. Katuli, R. Anousheh, S. Knutsen, P. Herring, J. Fan, Vegetarian diets and cardiovascular risk factors in black members of the Adventist Health Study-2, Public Health Nutrition FirstView (2014) 1-9.
[12] Y. Tantamango-Bartley, K. Jaceldo-Siegl, J. Fan, G. Fraser, Vegetarian diets and the incidence of cancer in a low-risk population, Cancer Epidem Biomar 22(2) (2013) 286-94.
[13] S. Tonstad, K. Stewart, K. Oda, M. Batech, R.P. Herring, G.E. Fraser, Vegetarian diets and incidence of diabetes in the Adventist Health Study-2, Nutrition, Metabolism and Cardiovascular Diseases 23(4) (2013) 292-299.
[14] S. Thomas, J. Reading, R.J. Shephard, Revision of the Physical Activity Readiness Questionnaire (PAR-Q), Canadian journal of sport sciences = Journal canadien des sciences du sport 17(4) (1992) 338-45.
[15] A. Bandura, Health promotion by social cognitive means, Health Educ Behav 31(2) (2004) 143-64.
[16] E. Morin, I. Michaud-Letourneau, Y. Couturier, M. Roy, A whole-food, plant-based nutrition program: Evaluation of cardiovascular outcomes and exploration of food choices determinants, Nutrition (Burbank, Los Angeles County, Calif.) 66 (2019) 54-61.
[17] N. Wright, L. Wilson, M. Smith, B. Duncan, P. McHugh, The BROAD study: A randomised controlled trial using a whole food plant-based diet in the community for obesity, ischaemic heart disease or diabetes, Nutrition & diabetes 7(3) (2017) e256.
[18] NHLBI. Your Guide to Lowering Cholesterol with Therapeutic Lifestyle Changes (TLC). NIH Publication No. 06–5235., (2005).

[19] Oldways African Heritage Diet Resources. http://oldwayspt.org/resources/heritage-pyramids/african-diet-pyramid. Accessed October 1, 2013.
[20] M.B. Bjerke, R. Renger, Being smart about writing SMART objectives, Evaluation and program planning 61 (2017) 125-127.
[21] K.L. Piercy, R.P. Troiano, R.M. Ballard, S.A. Carlson, J.E. Fulton, D.A. Galuska, S.M. George, R.D. Olson, The Physical Activity Guidelines for Americans, Jama 320(19) (2018) 2020-2028.
[22] DPP, The Diabetes Prevention Program (DPP), Diabetes Care 25(12) (2002) 2165-2171.
[23] A.F. Subar, S.I. Kirkpatrick, B. Mittl, T.P. Zimmerman, F.E. Thompson, C. Bingley, G. Willis, N.G. Islam, T. Baranowski, S. McNutt, N. Potischman, The Automated Self-Administered 24-Hour Dietary Recall (ASA24): A Resource for Researchers, Clinicians, and Educators from the National Cancer Institute, Journal of the Academy of Nutrition and Dietetics 112(8) (2012) 1134-1137.
[24] P.S. Freedson, E. Melanson, J. SIRARD, Calibration of the Computer Science and Applications, Inc. accelerometer, Medicine and Science in Sports and Exercise 30(5) (1998) 777-781.
[25] M. Hagstromer, P. Oja, M. Sjostrom, The International Physical Activity Questionnaire (IPAQ): a study of concurrent and construct validity, Public Health Nutr 9(6) (2006) 755-62.
[26] C. Mason, P.T. Katzmarzyk, Effect of the Site of Measurement of Waist Circumference on the Prevalence of the Metabolic Syndrome, The American Journal of Cardiology 103(12) (2009) 1716-1720.
[27] S.D. Ball, T.S. Altena, P.D. Swan, Comparison of anthropometry to DXA: a new prediction equation for men, European Journal of Clinical Nutrition 58(11) (2004) 1525-31.
[28] Z. Wang, S.B. Heymsfield, Z. Chen, S. Zhu, R.N. Pierson, Estimation of percentage body fat by dual-energy x-ray absorptiometry: evaluation by in vivo human elemental composition, Physics in Medicine and Biology 55(9) (2010) 2619-35.
[29] T.G. Pickering, J.E. Hall, L.J. Appel, B.E. Falkner, J. Graves, M.N. Hill, D.W. Jones, T. Kurtz, S.G. Sheps, E.J. Roccella, Recommendations for Blood Pressure Measurement in Humans and Experimental Animals: Part 1: Blood Pressure Measurement in Humans: A Statement for Professionals From the Subcommittee of Professional and Public Education of the American Heart Association Council on High Blood Pressure Research, Circulation 111(5) (2005) 697-716.
[30] L.V. Moore, A.V. Diez Roux, J.A. Nettleton, D.R. Jacobs, Associations of the Local Food Environment with Diet Quality—A Comparison of Assessments based on Surveys and Geographic Information Systems: The Multi-Ethnic Study of Atherosclerosis, American journal of epidemiology 167(8) (2008) 917-924.
[31] E. Cerin, B.E. Saelens, J.F. Sallis, L.D. Frank, Neighborhood Environment Walkability Scale: validity and development of a short form, Med Sci Sports Exerc 38(9) (2006) 1682-91.
[32] J.F. Sallis, R.B. Pinski, R.M. Grossman, T.L. Patterson, P.R. Nader, The development of self-efficacy scales for healthrelated diet and exercise behaviors, Health Education Research 3(3) (1988) 283-292.
[33] N.D. Barnard, L. Gloede, J. Cohen, D.J. Jenkins, G. Turner-McGrievy, A.A. Green, H. Ferdowsian, A low-fat vegan diet elicits greater macronutrient changes, but is comparable in adherence and acceptability, compared with a more conventional diabetes diet among individuals with type 2 diabetes, Journal of the American Dietetic Association 109(2) (2009) 263-72.
[34] N.D. Barnard, A.R. Scialli, P. Bertron, D. Hurlock, K. Edmonds, Acceptability of a therapeutic low-fat, vegan diet in premenopausal women., Journal of Nutrition Education 32(6) (2000) 314-319.
[35] N.D. Barnard, A.R. Scialli, G. Turner-McGrievy, A.J. Lanou, Acceptability of a low-fat vegan diet compares favorably to a step II diet in a randomized, controlled trial, J Cardiopulm Rehabil 24(4) (2004) 229-35.
[36] S. Cohen, T. Kamarck, R. Mermelstein, A global measure of perceived stress, Journal of health and social behavior 24(4) (1983) 385-96.

[37] C. Mushi-Brunt, D. Haire-Joshu, M. Elliott, Food spending behaviors and perceptions are associated with fruit and vegetable intake among parents and their preadolescent children, J Nutr Educ Behav 39(1) (2007) 26-30.
[38] J. Ware, Jr., M. Kosinski, S.D. Keller, A 12-Item Short-Form Health Survey: construction of scales and preliminary tests of reliability and validity, Medical care 34(3) (1996) 220-33.
[39] U.S. Adult Food Security Survey Module. https://www.ers.usda.gov/topics/food-nutrition-assistance/food-security-in-the-us/survey-tools.aspx#adult. Accessed July 3, 2019.
[40] A.J. Stunkard, S. Messick, The three-factor eating questionnaire to measure dietary restraint, disinhibition and hunger, J Psychosom Res 29(1) (1985) 71-83.
[41] M.R. Lowe, M.L. Butryn, E.R. Didie, R.A. Annunziato, J.G. Thomas, C.E. Crerand, C.N. Ochner, M.C. Coletta, D. Bellace, M. Wallaert, J. Halford, The Power of Food Scale. A new measure of the psychological influence of the food environment, Appetite 53(1) (2009) 114-118.
[42] A. Steckler, L. Linnan, Process evaluation for public health interventions and research, Jossey-Bass, San Francisco, 2002.
[43] R.P. Saunders, M.H. Evans, P. Joshi, Developing a process-evaluation plan for assessing health promotion program implementation: a how-to guide, Health promotion practice 6(2) (2005) 134-47.
[44] Y. Ma, B.C. Olendzki, J. Wang, G.M. Persuitte, W. Li, H. Fang, P.A. Merriam, N.M. Wedick, I.S. Ockene, A.L. Culver, K.L. Schneider, G.-F. Olendzki, J. Carmody, T. Ge, Z. Zhang, S.L. Pagoto, Single-Component Versus Multicomponent Dietary Goals for the Metabolic SyndromeA Randomized TrialComparison of High-Fiber and AHA Diets, Annals of Internal Medicine 162(4) (2015) 248-257.
[45] N.M. Mirza, M.G. Palmer, K.B. Sinclair, R. McCarter, J. He, C.B. Ebbeling, D.S. Ludwig, J.A. Yanovski, Effects of a low glycemic load or a low-fat dietary intervention on body weight in obese Hispanic American children and adolescents: a randomized controlled trial, The American Journal of Clinical Nutrition 97(2) (2013) 276-285.
[46] N.D. Barnard, S.M. Levin, Y. Yokoyama, A Systematic Review and Meta-Analysis of Changes in Body Weight in Clinical Trials of Vegetarian Diets, Journal of the Academy of Nutrition and Dietetics 115(6) (2015) 954-969.
[47] N.D. Barnard, J. Cohen, D.J. Jenkins, G. Turner-McGrievy, L. Gloede, A. Green, H. Ferdowsian, A low-fat vegan diet and a conventional diabetes diet in the treatment of type 2 diabetes: a randomized, controlled, 74-wk clinical trial, Am J Clin Nutr 89(5) (2009) 1588S-1596S.
[48] N.D. Barnard, J. Cohen, D.J. Jenkins, G. Turner-McGrievy, L. Gloede, B. Jaster, K. Seidl, A.A. Green, S. Talpers, A low-fat vegan diet improves glycemic control and cardiovascular risk factors in a randomized clinical trial in individuals with type 2 diabetes, Diabetes Care 29(8) (2006) 1777-83.
[49] M.L. Toohey, M.A. Harris, W. DeWitt, G. Foster, W.D. Schmidt, C.L. Melby, Cardiovascular disease risk factors are lower in African-American vegans compared to lacto-ovo-vegetarians, J Am Coll Nutr 17(5) (1998) 425-34.
[50] G.M. Turner-McGrievy, N.D. Barnard, J. Cohen, D.J. Jenkins, L. Gloede, A.A. Green, Changes in nutrient intake and dietary quality among participants with type 2 diabetes following a low-fat vegan diet or a conventional diabetes diet for 22 weeks, Journal of the American Dietetic Association 108(10) (2008) 1636-45.
[51] G.M. Turner-McGrievy, M.D. Wirth, N. Shivappa, E.E. Wingard, R. Fayad, S. Wilcox, E.A. Frongillo, J.R. Hébert, Randomization to plant-based dietary approaches leads to larger short-term improvements in Dietary Inflammatory Index scores and macronutrient intake compared with diets that contain meat, Nutrition research (New York, N.Y.) 35(2) (2015) 97 - 106.
[52] M.A. Cotton, C. Ball, P. Robinson, Four simple questions can help screen for eating disorders, J Gen Intern Med 18(1) (2003) 53-6.
[53] H.K. Seligman, B.A. Laraia, M.B. Kushel, Food insecurity is associated with chronic disease among low-income NHANES participants, The Journal of nutrition 140(2) (2010) 304-310.

Table 1: Inclusion and exclusion criteria for the Nutritious Eating with Soul (NEW Soul) study

Inclusion criteria	Exclusion criteria
self-identify as African American	currently following a vegan diet
18-65 years of age	pregnant (or have been pregnant in the last 6 months), anticipating on becoming pregnant in the next 24 months, or currently breastfeeding
BMI between 25- 49.9 kg/m^2	current participation in a weight loss program or taking weight loss medications (although participants may be trying to lose weight on their own)
live in the Columbia, SC area	recent weight loss (>10 lbs in the last 6 months)
be able to attend all monitoring visits	has type 2 diabetes that is controlled with medications (vs. controlled with diet and exercise)
be willing to be randomized to either diet	has an uncontrolled thyroid condition
be free of major health or psychiatric diseases, drug or alcohol dependency	
be free of an eating disorder as screened by the Eating disorder Screen for Primary care [ESP] [52]	
be free on either of the two meeting nights (e.g., Monday or Wednesday)	

Table 2: Measurements collected at each time point in the NEW Soul study

**Study Year**	**Year 1** Months				**Year 2** Months			
**Questionnaire measures**	0	3	6	12	15	18	21	24
Demographic Information	X							
Perceived Stress Scale [36]	X	X	X	X				X
Short Form Health Survey (SF-12) [38]	X	X	X	X				X
Self-Efficacy for Diet Behaviors [32]	X	X	X	X				X
U.S. Adult Food Security Survey Module [53]	X	X	X	X				X
Food Spending Items (adapted from [37])	X	X	X	X				X
Three-Factor Eating Questionnaire [40]	X	X	X	X				X
Power of Food Scale [41]	X	X	X	X				X
Dietary Intake (ASA-24) [23]	X	X	X	X				X
International Physical Activity Questionnaire (IPAQ) short version [25]	X	X	X	X				X
Current Medications	X		X	X				X
Dietary Adherence and Acceptability [33-35]		X	X	X				X
**Lab-based assessments**	0	3	6	12	15	18	21	24
Height and Weight	X		X	X				X
Physical Activity (ActiGraph Accelerometer GT1M model)	X		X	X				X
Blood Pressure	X		X	X				X

Fasting Lipids, Glucose, and<br><br>Insulin	X		X	X				X
Body Fat (DEXA Scan)	X			X				X
Waist-to-Hip Circumference	X		X	X				X

Table 3: Dietary recommendations for the NEW Soul study participants randomized to follow the Vegan or Omnivorous diets

**Dietary Guidelines**	**Vegan**	**Omnivorous**
Grains	Focus on whole grains with at least 3 g of fiber per serving.	Eat 6 or more servings of grains and make most of your grains whole grains.
Vegetables	Ensure that 20-50% of your plate should be vegetables, including leafy greens.	Eat 3-5 servings of vegetables a day.
Legumes	Eat 1-1½ cups of legumes a day (beans, peas or lentils).	Eat ½-1 cup of legumes a day (beans, peas or lentils).
Fruit	Eat 2-4 servings of fruit each day.	Eat 2-4 servings of fruit each day.
Vegetable oils/Essential fatty acids	Avoid vegetable oils. Eat 1 tablespoon of ground flax seed, hemp or chia seeds every day for omega-3 fatty acids.	Use small amounts of healthy oils, like sesame or olive oil for dressings, and canola oil for cooking.
Nuts/seeds	Eat high fat plant foods (e.g., avocados, olives, coconut, nuts, seeds and nut butters like peanut butter) as condiments to flavor a meal or as a recipe ingredient, but not a snack.	Add nuts and seeds to at least one meal a day or eat as a snack.
Treats	Limit treats to one time per month. Treats are foods that are higher in fat and sweeteners than the recommendations provide.	Limit treats to one time per month. Treats are foods that are higher in fat and sweeteners than the recommendations provide.

Beverages	Avoid drinking liquid calories.	Avoid drinking liquid calories.
Meat, fish, poultry, eggs	Avoid all meat, fish, poultry, and eggs.	Eat no more than 3-5 ounces of lean meat per day and no more than 2 egg yolks per week.<br>• In addition, eat two servings of fish per week.
Dairy	Avoid all dairy.	Consume dairy in small portions, and if you are lactose intolerant, enjoy other calcium-rich foods like greens, beans, and almonds.<br>• Consume 2-3 servings of low- or no-fat dairy products a day.<br>• Cheese should have no more than 3g of fat per ounce.
Sodium and seasonings	Limit use of sodium and use herbs and spices to flavor your food.	Limit use of sodium and use herbs and spices to flavor your food.
Vitamin B12	Take a B12 vitamin daily.	Take a B12 vitamin daily.
Alcohol	Limit or avoid alcohol: If you do drink, have no more than one drink a day for women and two a day for men.	Limit or avoid alcohol: If you do drink, have no more than one drink a day for women and two a day for men.

Table 4: Class structure for the NEW Soul study and Social Cognitive Theoretical constructs targeted

**Order and structure of each NEW Soul class**	**Social Cognitive Theory constructs targeted**
Review of SMART goal from previous week	Self-efficacy
Group problem solving of challenges from the week	Facilitation
Addressing stress and management techniques for stress	Emotional Coping Responses
Cooking demonstration (or hands-on cooking class)	Observational Learning (or Behavioral Capability)
Prize drawing for those who attend class each week	Incentive Motivation
Setting of SMART goal related to the topic of the week	Self-regulation

Table 5: Class topics for the Nutritious Eating with Soul (NEW Soul) Study

Class Number	Class Topics*
**Weekly classes**	
Class 1	Orientation & Introduction to the NEW Soul Study/Breakfast Ideas
Class 2	Basic Nutrition Guidelines/Lunch Ideas
Class 3	Dinner Ideas
Class 4	Batch Cooking
Class 5	Motivation - What is your WHY?
Class 6	Making a Meal Plan
Class 7	Grocery Store Tour
Class 8	Movement and Physical Activity
Class 9	Greens
Class 10	Carbohydrates & Whole Grains
Class 11	Volumetrics
Class 12	Protein
Class 13	Tips for Dining Out and Traveling
Class 14	Nuts/Seeds/Fats
Class 15	Tubers and Starchy Vegetables/Fiber
Class 16	Recipe Redux: Making Over Family Favorite Recipes
Class 17	Mind the Salt
Class 18	Fruits and Vegetables
Class 19	Emotional Eating

Class 20	Mock Meats - Beef/Burgers
Class 21	Social Support
Class 22	Desserts
Class 23	Snacks
Class 24	Celebration: The Six-month Mark
**Bi-weekly classes**	
Class 25	Back to the WHY: Keeping your Motivation
Class 26	Stress Management
Class 27	Religion and Diet: Part 1
Class 28	Inflammation
Class 29	Holiday Tips
Class 30	Reading Menus and Meal Planning
Class 31	Diabetes & Carbohydrates
Class 32	Chopped!
Class 33	Fad Diets
Class 34	Staying on Track
Class 35	Cholesterol
Class 36	Setting Goals
**Monthly classes**	
Class 37	Frozen Food Taste Test
Class 38	Religion and Diet: Part 2
Class 39	Dementia and Diet
Class 40	Massage for Stress Management and Meal Planning

Class 41	Appetizers
Class 42	Diet and the Environment
Class 43	Cooking/Storage Tips
Class 44	Physical Activity
Class 45	Being a Savvy Shopper
Class 46	Holiday Planning
Class 47	Summary and Reflections
Class 48	Conclusion & Certificate of Completion

*Class topics for Cohort 2 were updated throughout the study, although the order and content largely remained the same

Adjustments to Laboratory Assessments due to COVID-19

In response to the threat of COVID-19 on the health of the public, an executive order from the South Carolina Governor's office was issued and all system institutions of the University of South Carolina were closed beginning March 16, 2021. University IRB approval was granted to resume in-person research based on established protocol set forth by NEW Soul supervisory staff for workers and participants. Workers hired to conduct assessments were tested for COVID-19 and were trained both virtually and in-person on safety measures and social distancing protocol. Staff training included proper handwashing and mask wearing. Additionally, staff was trained on work shift check-in procedures that included a temperature check and answering COVID-19 screening questions prior to each shift to assess potential illness and COVID-19 exposure. Staff were also trained on participant interaction and appointment check-in. Participants also received a detailed account of safety measures in place via personal phone calls, email and text message. Table 1 details which cohorts and timepoints were impacted by COVID-19 and Table 1 details which measures were impacted by COVID-19.

Table 1: Cohorts and measurement timepoints impacted by COVID-19

**Impacted by COVID?**	**Cohort 1**	**Cohort 2**
**6-month assessments**	No	No
**12-month assessments**	No	Yes
**24-month assessments**	Yes	Yes

Table 2: Summary of All Measures Being Collected at 12 and 24-month assessments and alterations due to COVID-19

**Cohort**	**Cohort 1 12-month**	**Cohort 2 12-month**	**Cohort 1 24 month**	**Cohort 2 24 month**
**Calendar month and year**	April 2019	June-July 2020	July-August 2020	April-May 2021
**Measure**	**Collected (x) or Not collected (---)**			
Weight	x	x	x	x
Physical Activity (ActiGraph Accelerometer GT1M model)	x	---	---	x
Blood Pressure	x	x	x	x

Bloodwork (Fasting Lipids, Glucose, and Insulin)	x	x	x	x
Body Fat (DEXA Scan)	x	x	---	x
Waist-to-Hip Circumference	x	---	---	x

ASSESSMENT GUIDE FOR PARTICIPANTS TO MITIGATE THE RISK OF COVID-19 WHEN COMPLETING ASSESSMENTS

- **A screening process to determine the COVID-19 risk status of volunteers and staff (e.g. questionnaire regarding health status, recent travels, body temperature, etc.).**
  Upon arrival, participants will sit in their car and call the number sent to them via the instruction sheet. A staff member will respond and ask them questions regarding health status, travel, and recent exposures. This staff member will also take their temperature before allowing them in the facility.

- **Procedures for:**
  - **maintaining social distancing requirements**
    All participants will wait in their car to avoid any overlap with other participants. All participants and staff will be wearing a mask and gloves. Each participant will be working with one specific staff member to minimize contact.

  - **use of PPE, including what is required and how it will be obtained**
    All participants will be wearing a face mask and gloves that are provided by the study and will be asked to wear them prior to entering the facility and remain wearing them at all times. Hand sanitizer will be provided throughout the facility. All staff members will also be wearing a face mask and gloves. New gloves will be used between participants.

  - **proper cleaning of materials, equipment and commonly touched areas where the activities will take place**
    Each room will be sanitized between participants. Staff will use new gloves between participants. All surfaces will be wiped down with disinfectant wipes. Extra attention will be paid to commonly touched surfaces like doorknobs, handles, and chairs. Participants and staff will not be allowed to eat or drink in the facilities. Bathroom use will be minimized, and the bathroom will be sanitized between use. Participants should be discouraged from touching surfaces; staff should do things like open and hold doors if possible.

  - **plan for scheduling visits to avoid overlap in appointments**
    All participants will be sign up for a specific time in advance. All participants must arrive at their designated time and will be asked to wait in their vehicle until we are ready for them to enter the

facility, one participant at a time. No one will be allowed to enter a room where another participant is currently located or before it has been sanitized.

- **if included in the subject population, plans for handling visits by participants in high-risk categories (older adults and anyone with serious underlying medical conditions). Refer to CDC guidance for a full list.**
  All participants with underlying health conditions will be instructed to contact us in advance. We will provide them with a list of conditions to notify us about. Current procedures should be enough but knowing which participants to specifically look out for will allow us to be extra cautious.

MANUAL OF OPERATING PROCEDURES

2017-2021

Version Date 5-10-2021

Table of Contents

PURPOSE **5**

Significance and Rationale........ 5

Study Aims........ 5

METHODS & PROCEDURES **6-21**

Overview of Intervention........ 6

Recruitment........ 6

Inclusion Criteria........ 7

Exclusion Criteria 7

Guidelines for Interacting with Potential Participants 8

Incoming Calls from Potential Participants 9

Incoming Emails from Potential Participants 10

Screening Process 11

Contacting Participants by Phone to Complete Phone Screener........ 12

Following-up from Phone Screener Unanswered Calls 12

Ineligible Participants........13-14

Eligible Participants........15

Physician Consent 16

Pre-Orientation Survey........17

Confirmation of Pre-Orientation Survey........18

Orientation Session 19-22

INTERVENTION ACTIVITIES **23-28**

Guidelines for Interacting with Participants 23

Consultants........ 24

Overview of Classes........24-25

Procedures for Classes........26-27

Kitchen Crew Tasks........28

Weekly Emails, Social Media Group and Audio Podcasts, Noncompliant Participants, Participant Expectations for Physical Activity, Treatment Fidelity Checks........29

LAB ASSESSMENTS........**30-47**

Participant Instructions to Prepare for your Lab Assessment & Assessment Reporting 30

Procedures for Signing-up Participants for 6, 12, and 24 Month Lab Assessments........31

Lab Assessment Reminders to Participants……………………….……………….……………………...30
Operation Procedures for Lab Assessments…………………......………………………………32-34
Procedures for Blood Pressure………………………………………………………….........35
Procedures for Height ……………………..……………………………………………………...36
Procedures for Weight ……………………..……………………………………………………..37
Procedures for Waist Circumference…..……………………………………………………………38
Procedures for Hip Circumference…..……………………………………………………...39
Managing Multiple Participants During Assessments…………………………………………………40
Participant Instructions for Dressing for DEXA Scan…………………………………………….....40
Procedures After Collection of Measurement and Check-out…………………………………………41
Procedures for Randomization of Diet Groups after Baseline…..……………………………………42
Procedures for Distributing Incentives at 3,6,12 and 24 Month Assessments………………………..42
Procedures for Lab Assessments at 3 months…………………………………………………………43
Procedures for Serum Preparations……………………………………………………………………43
Procedures for Plasma Preparations…………………………………………………………...44
Procedures for Participants Whose Samples Can't be Collected by CERC…………………………...45
MEASUREMENTS………………………………………………………………………………46-51
Summary of all Measures Being Collected……………………………………………………...46
Procedures for Dietary Recalls…………………………………………………………………..47
Email Link to Complete ASA-24……………………………………………………………48-49
Tracking the Recall………………………………………………………………………......50-51
Incomplete Dietary Recalls……………………………………………………………………………51
Common Technical Problems with the ASA-24………...……………………………………………51
Procedures for Accelerometers to Track Physical Activity…........……………………………………...52
DATA MANAGEMENT
Collection and Storage ………………...………………………………………………………...53
Tracking Data………………………...………………………………………………………….53
Study Grant Application………………………………………………………………………….53
Study Staff and Personnel ……………………………………………………………………..54
Additional Forms for Student Staff/Volunteers…………………………………………………………55

Guidelines for Dietary Adherence Calls with Participants……………………………………………..56-57

LITERATURE CITED…………………………………………………………………….......58-59

PURPOSE

Significance and Rationale

The overall goal of this study is to assess the impact of different dietary patterns on cardiovascular disease (CVD) risk factors among African American (AA) adults. The study will test two culturally-tailored soul food diets in a two year randomized controlled trial: a vegan (plant-based) diet or a low-fat omnivorous (omni) diet.

Plant-based diets (PBDs), which include vegan diets, lactovegetarian diets, pesco-vegetarians diets, or semi-vegetarian diets, are associated with a lower risk for certain chronic diseases, such as diabetes, cardiovascular disease, and some cancers compared to the typical Western diet.[1-6] PBDs are also associated with a lower Body Mass Index (BMI),[7,8] improved sleep quality,[9,10] and healthier mood states.[11,12] While numerous observational studies and clinical trials have consistently demonstrated health benefits from consuming a PBD,[13] only a few of them have focused on ethnic minorities.[2,14] There is a need for intervention research to assess potential health benefits from consuming a PBD among AAs and to find ways to make interventions culturally-relevant by including specific plant-based foods that have a historic and meaningful cultural context in the AA community (e.g. soul food in the South originates from a mostly plant-based, West African diet).[15,16] This study will address gaps in the PBD literature by being the first randomized trial to focus on AAs, a traditionally understudied population in nutrition research.

Study Aims

Primary Aims: Using a randomized design, determine the impact at 12 months of two different, culturally tailored diets (n = 65 omnivores and n = 65 vegan) on changes in:

1. **Risk factors for CVD, including LDL cholesterol and blood pressure; and**
2. **Body weight.**

a. <u>Hypothesis for Aims 1 and 2</u>: We hypothesize that CVD risk factors and body weight will improve to a greater extent in the vegan group as compared with the omni group.

Secondary Aim:

3. **Examine long-term changes in CVD risk factors and body weight at 24 months.**

a. <u>Hypothesis for Aim 3</u>: We hypothesize that the greatest improvements in CVD risk factors and body weight will be seen within the vegan group as compared with the omni group, demonstrating maintenance of health-related behaviors

METHODS AND PROCEDURES

Overview of Intervention

The intervention is a two-year, two-arm, individually randomized clinical trial comparing two different dietary patterns (vegan and omnivorous diets) on changes in CVD risk factors and body weight among overweight or obese AA adults. Participants will participate in weekly group classes for six months, bi-weekly classes for the next six months, then monthly classes for the remaining 12 months. The classes will be led by dietary interventionists where participants will receive cooking lessons, recipe ideas, and guidance for adhering to their diet. Classes will be augmented with remotely-delivered support, including audio podcasts and Facebook groups. Both groups will receive cooking classes and recipes that are culturally-tailored to Southern cuisine. Participants will have several measurements, including demographic information, height and weight, physical activity, and dietary intake assessed at baseline, 3 months, 6 months, 12 months, and 24 months. Biometric measurements (blood work) will be assessed at baseline, 6 months, 12 months, and 24 months. Complete details for each measurement are discussed under the measurements subsection of this manual.

Recruitment

The goal is to recruit a total of 130 participants (65 participants for Cohort 1 and 65 participants for Cohort 2). Recruitment for Cohort 1 will begin in January of 2018. Recruitment for Cohort 2 will begin in January of 2019. Participants will be recruited from Columbia, SC and nearby communities via the media (print, television, web and radio), promotional materials (i.e. fliers and brochures), targeted community outreach efforts (i.e. neighborhoods, churches, libraries, employers, recreation commissions and coalitions), word of mouth and mass mailings. Participants will be directed to the study's website to learn more information and complete a screening questionnaire.

Inclusion Criteria (assessed from online screening survey and/or telephone screening conversation):

- Self-identify as African American.
- Be 18-65 years of age.
- Have a BMI between 25- 49.9 kg/m^2.
- Live in Columbia, SC or a surrounding area.
- Be free on either of the two meeting nights for classes (e.g., Monday or Tuesday).
- Be able to attend all monitoring visits.
- Be willing to be randomized to either diet group.
- Not currently following a vegan diet.
- Be free of major health or psychiatric diseases, drug or alcohol dependency, thyroid conditions, diabetes, or pregnancy.

Exclusion Criteria (assessed from online screening survey and/or telephone screening conversation):

- Women that are pregnant, have been pregnant in the last 6 months, anticipate becoming pregnant in the next 24 months, or currently breastfeeding.
  - Women who are pregnant should not be pursuing weight loss and should be under the direct care of a physician. Therefore, women who are pregnant or who are anticipating they might be pregnant should not participate in this study. If a woman becomes pregnant during the study, she will be advised to consult her care provider and will be dropped from the weight loss study.

- Someone with an eating disorder (which will be screened with the Eating disorder Screen for Primary care [ESP]).[17]
  - If a participant has an eating disorder, they will be given contact information for the eating disorder clinic at the University of South Carolina.

- Currently participating in a weight loss program or taking weight loss medications
  - (Participants can be included in the study if they are trying to lose weight on their own).

- Individual recently lost a significant amount of weight (>10lbs in the last 6 months).

Guidelines for Interacting with Potential Participants

- **Maintain a pleasant and friendly attitude in all conversations with participants to place them at ease.** This positive rapport with the participant will ensure the willingness of the participant to remain in the study.

- **Assure participants' confidentiality.** Confidentiality should be maintained throughout the study. Anytime a participant asks about confidentiality reassure him/her that data and information will be de-identified, meaning their names won't be used for during data analysis and any document with one's name (i.e. consent forms) will be stored in a locked cabinet that only the research team will have access to. Other aspects of confidentiality to consider are not discussing any names or information outside of the study setting, making sure that written surveys are collected responsibly and not left in sight for anyone else to see them, and not discussing participants' medical records with anyone.

- **Be respectful**. This includes being punctual to study participants' classes, lab visits and measurements. To avoid unexpected delays, it is recommended that study personnel should arrive at the meeting locations at least 15 minutes prior to the appointment time. During measurement sessions, study staff should turn off personal cell phones. Study personnel should also maintain professional appearance, dress and speech. Additionally, we are working with overweight or obese African American participants and study personnel should be sensitive and respectful about culture and issues related to body weight and image. Jokes or sarcasm relating to African American culture, weight or body image should be avoided.

Incoming Calls from Potential Participants

Persons will call in after hearing about the study and request additional information. Please see script below.

Note: Please also leave the script below on your voicemail. This will cut down the amount of return calls requested due to needing more information.

Script

Thank you for your interest in the NEW Soul Study! This study is a **24-month intervention** examining how a plant-based, soul food diet and a low-fat, standard, soul food diet, may help improve heart disease risk factors and nutrition in the African American community, and help with weight loss. The study includes…

- nutrition and cooking classes
- access to a Nutritionist
- group support
- up to $210 in financial compensation
- recipes and more

In order to qualify to participate you must:

- Self-identify as African American
- Be between the ages of18-65 years
- Body Mass Index between 25- 49.9 kg/m2
- Live in the Columbia, SC/Midlands area
- Be able to attend all monitoring and weekly class visits
- Be willing to be randomized to either diet
- Not currently following a vegan diet
- Not currently on medication for diabetes
- Not currently pregnant or breastfeeding (or plan to become pregnant in the next 24 months)

If you meet these qualifications, please visit our website at newsoul.org (spell it out as well) and click on "Participate" and then click on "Take the Screener". This will lead you to a short questionnaire that you will complete online. Upon completion, please allow for up to a week for our staff to contact you regarding your eligibility.

Incoming Emails from Potential Participants

Note: You will receive online inquiries about the study through Brie.net or the study's "contact us" page. Below is a sample email response.

Hi, (insert name)!

Thank you for expressing interest in the Nutritious Eating With Soul (NEW) Study!

The NEW Soul Study is a clinical trial funded by the National Institutes of Health based on the alarming statistics below.

In the US, African Americans (AA) have the highest rates of obesity and heart disease as compared with whites and Hispanics.

More AA men and women die from cardiovascular disease (CVD) than any other chronic disease condition.

Overweight and obesity are associated with a number of chronic diseases, including type 2 diabetes, hypertension and CVD, as well as an increased risk of several cancers.

NEW Soul can help.

Participating in the NEW Soul Study can help you improve your blood pressure, cholesterol levels, and lose weight while eating soul food.

Next Steps

Please visit our website, www.newsoul.org for additional information and to take the online screener located on the "Participate" page. Once you complete this, a member of our staff will contact you.

We look forward to receiving your information!

Screening Process

Note: Study Coordinators participating in the screening process must be trained by the Project Manager. This training will include observation of phone screenings conducted by experienced staff and being observed by the Project Manager or experienced staff in conducting a phone screening.

1. Potential Participants must complete the online screener on newsoul.org.
    a. The specified Study Coordinator will download all responses into the TRACKING google excel sheet on google drive onto the "need to screen" tab.
    b. Upon downloading all responses from the online screener, the specified Study Coordinator should send an email confirming receipt of the screener and providing information on what to expect. See below email.

> Greetings!
>
> Thank you for your interest in the NEW Soul Study and completing the online screener! We have received your completed online screener and your information is under review. Please be on the lookout for an email or phone call within the next week from a member of our team regarding your eligibility to participate in the study. Thank you for your patience and you will hear from us soon.

    c. The specified Study Coordinator will review all responses and cut and copy the information to the appropriate tab of the TRACKING sheet.
    d. The Project Manager will review all responses on the "Uncertain to include and exclude" tab, consult with the PI as needed, and cut and copy the information to the appropriate tab.

2. Within a week of the online screener completion date, eligible persons will be contacted to complete the phone screener; this includes signing up to attend orientation. Ineligible persons will receive an email informing them on their ineligibility.

Contacting Potential Participants to Complete Phone Screener

Participants should be first screened from the online survey and then study personnel will do the phone screener. When calling a participant that qualifies for the study, introduce yourself and ask them if they will be interested in enrolling in the study. Give the time and date for the orientation meeting and answer any questions that they might have. An introductory phone call may sound like this:

Hello my name is _______. I am a study coordinator at the University of South Carolina for the NEW Soul study. I am contacting you because you recently filled out our online screening questionnaire and I would like to ask you some additional questions and answer any questions you may have. This will take approximately 10-15 minutes of your time. Is now a good time to talk? If no, "What is a good day and time to call you back? If yes, "Wonderful, I have a few personal questions to ask regarding your eligibility to participate in the NEW Soul Study. Please note that your responses will only be accessible by NEW Soul Staff for eligibility purposes. We will not share your information with any outside persons or parties. Does this sound okay? (pause). Let's proceed.

Link to complete phone screener: http://www.surveygizmo.com/s3/3926626/NEW-Soul-Study-Phone-Screener

Note: Follow the scripts listed throughout the phone screener.

Following-up from Unanswered Calls when attempting to complete Phone Screener

Some participants may not answer their phones, especially from numbers that do not have an 803 area code. Leave a voice message explaining who you are and that you were calling regarding their eligibility for the NEW Soul Study. Leave a call back number and repeat the call back number twice and very slowly. Wait 24 hours for them to call back. If they have not called by then, try and do a second phone call. This should all be tracked in the google document "TRACKING SHEET". An email that includes the above information should be sent to participants who you are not able to leave a voicemail for or to a participant who can not be reached after a second unsuccessful phone attempt. Sample email below.

> Hi, (insert name of participant)-
>
> My name is _______, and I am a study coordinator at the University of South Carolina for the NEW Soul study. I am emailing you because you recently filled out our online screening questionnaire and I have not been able to reach you by phone to complete the second step in the screening process. The phone screening will include some additional questions about your interest and ability to participate in the study and will take approximately 10-15 minutes of your time. Please let me know a good day and time to call you back or you can give me a call back at (insert number) between the hours of (insert availability) on (insert days available) . I look forward to hearing from you soon!
>
> (insert email signature)

Ineligible Participants

The reasons participants aren't eligible to enroll in the study should be tracked in the excel "TRACKING SHEET". Below are the reasons for ineligibility.

Exclusion Responses

**1**	**Does not identify as African American**
**2**	**Does not meet the age requirements**
**3**	**Does not meet the BMI requirements**
**4**	**Lost more than 10 pounds in the last 6 months** (flexible on this, see Project manager or PI)
**5**	**Unable to attend all classes**
**6**	**Not willing to be randomized to a diet group**
**7**	**Currently following a vegan diet**
**8**	**Not willing to be vegan for two years**
**9**	**Taking medication for diabetes**
**10**	**Currently pregnant, breastfeeding, or plan to become pregnant in the next 24 months**
**11**	**Other**

***Additionally, anyone who has had a heart attack or stroke is ineligible. You will also want to check with PI regarding any disease diagnosis.**

Date	Reason for Ineligibility	Reason for Not Participating
4/27/2015		Unwilling to be randomized
4/27/2015	High BMI ($\geq$ 50)	
4/29/2015	Normal BMI (< 25)	

Example of tracking the reasons participants were ineligible for the study

All <u>ineligible</u> participants should receive the email below and document it in the tracking sheet.

Insert Date

Dear Interested Participant:

Thank you for your interest in the NEW Soul Study conducted at the University of South Carolina. Based on the information you provided to us in the online screener, we regret to inform you that you are not eligible to participate in this study. Please understand that because this is a research study, we are seeking individuals that meet very specific criteria including the following: age, weight/height, weight history, medical history, and willingness to be randomized to follow either a vegan or low-fat omnivorous diet.

Again, we are sorry that we are unable to have you as a participant in our current study but hope that you will be able to participate in one of our programs in the future. If you have any questions regarding the questionnaire or the research program, please contact Mary Wilson via email at mjwilson@mailbox.sc.edu or via phone at 803.777.1902.

Sincerely,

The NEW Soul Study Team

Brie Turner-McGrievy, Ph.D., M.S., R.D.
Assistant Professor
Department of Health Promotion, Education, and Behavior
NEW Soul Study, Prinicipal Investigator
Arnold School of Public Health
University of South Carolina
915 Greene St., Columbia, SC 29208
Phone: 803-777-3932
Fax: 803-777-6290
E-mail: brie@sc.edu

Mary Wilson, M.P.H.
Project Manager
Department of Health Promotion, Education, and Behavior
Arnold School of Public Health
University of South Carolina
915 Greene St., Columbia, SC 29208
Phone: 803.777.1902
Fax: 803.777.6290

Eligible Participants

All eligible participants should receive the email below from the Project Manager.

Email Subject Line: Welcome to the NEW Soul Study!

Insert Date

Dear (Insert Participants Name):

Thank you for your interest in participating in the NEW Soul Study conducted at the University of South Carolina. Based on the information you provided to us in the online and phone screener, we are excited to inform you that you are eligible to participate in this study!

As a reminder, you signed up to attend orientation on (insert date) from (insert time). Please add this information to your personal calendar. You will also receive reminders from our office leading up to the date. The location for orientation is below.

Address:
Discovery I
915 Greene St., Room 140
Columbia, SC 29208

Please Note: We have received an overwhelming response rate from interested participants, so please be on the lookout for a future email in early March that will include a survey that you will need to complete to express that you are still interested in the study. This email will also include additional instructions regarding parking for orientation.

In the meantime, please follow us on social media.

Facebook: https://www.facebook.com/NEWSoulStudy/
Instagram: https://www.instagram.com/newsoulstudy/
Twitter: https://twitter.com/NEWSoulStudy

If you have any questions or need to reach me prior to your orientation date, please feel free to contact me via email or phone.

Sincerely,

Mary Wilson, M.P.H.
Project Manager, NEW Soul Study
Department of Health Promotion, Education, and Behavior
Arnold School of Public Health
University of South Carolina
915 Greene St., Columbia, SC 29208
Phone: 803.777.1902
Fax: 803.777.6290

Physician Consent

All eligible participants requiring physician consent should receive the email below immediately after completing the phone screener. Please mail to participant if requested. The date sent and the date received should be documented in the tracking sheet. Attach the NEW Soul Info Flyer and Physician Consent Form to the email. The physician consent form can be found here:

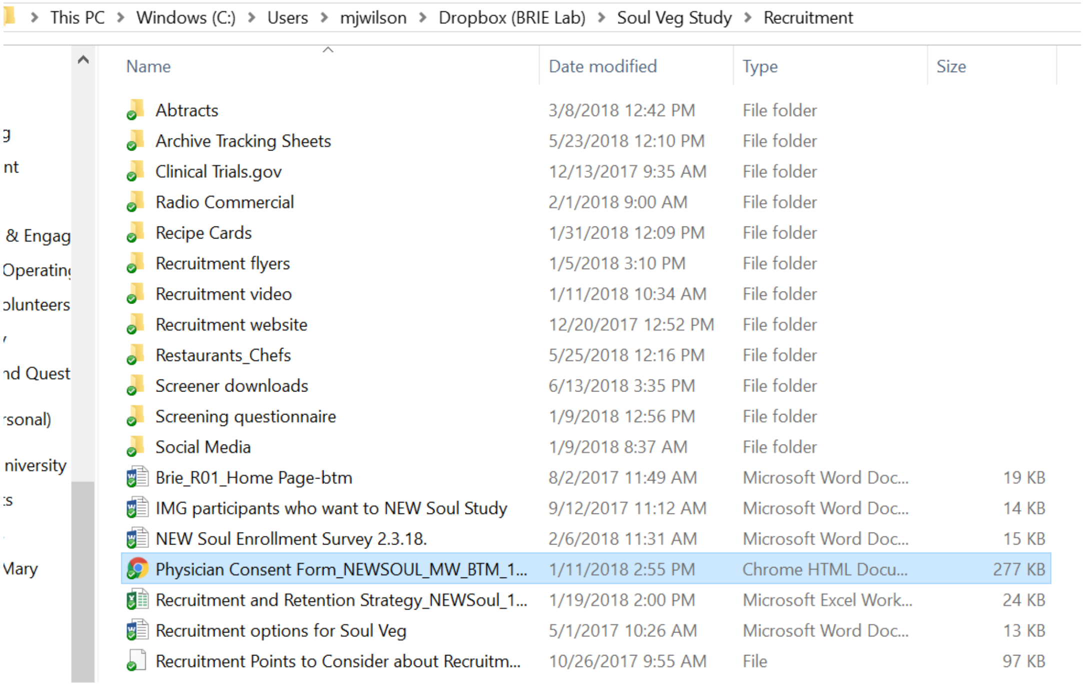

Good Morning/Afternoon (insert name of participant),

It was great speaking with you today! As discussed during your phone questionnaire, you will need to obtain physician consent prior to participating due to taking medication associated with hypertension/high blood pressure. Please have your physician complete the attached document and return to our Project Manager by (insert date that is two weeks prior to the first orientation session). Also, attached is a flyer that you can share with your physician. If faxing, please use a FAX Cover Sheet with attention to Mary Wilson, Project Manager of NEW Soul Study. Her contact information is included on the Physician Consent for. Please let me know if you have any questions and I look forward to working with you!

Kindest Regards,

(insert name)

Pre-Orientation Survey

All participants signed up for orientation will be sent the following email at the beginning of the month of the first orientation session. See email below. Emails will be sent according to the orientation date participant signed up for.

Email Subject Line: NEW Soul Pre-Orientation Survey (Response Required)

Insert Date

Dear (Insert Participants Name):

Dear NEW Soul Study Participant:

We are really excited to get started with the Nutritious Eating With (NEW) Soul Study and have had an overwhelming response from people who want to enroll in the study! Below are a few items that need your attention.

1. **REQUIRED SURVEY:** In order to ensure you are still interested in participating in this study, please **"CLICK HERE" to complete the survey to secure your spot or** you can follow the link here: **http://www.surveygizmo.com/s3/4180490/NEW-Soul-Study-Pre-Orientation-Survey.**
    a. **Please note that you are required to complete this survey by March 15th**. Upon completion of the survey, you will receive instructions for parking and access to our office building.

2. **ORIENTATION:** As a reminder, your orientation date is **(insert day of week, date, and time).**
3. **STUDY CONSENT:** Attached is a pdf copy of the consent form. Please read through this prior to orientation. You will have time to read and sign at orientation, but we want to make sure you have a good understanding of what will be covered before orientation.

If you have any questions, please feel free to contact Mary Wilson, Project Manager at 803.777.1902 or mjwilson@mailbox.sc.edu.

Thank you and we look forward to seeing you soon!

(insert name)

Confirmation of Pre-Orientation Survey

Upon receipt of the pre-orientation survey, all participants should receive the email below. The document that provides directions to the orientation location and parking instructions can be found in:

Dropbox (BRIE Lab) > Soul Veg Study > Orientation session > Communication Re Orientation

Name	Date modified	Type	Size
Discovery Directions_NEW Soul	5/7/2018 10:04 AM	Microsoft Word Doc...	537 KB
Invitation to Orientation_Mail Merge_NEW Soul...	2/6/2018 12:41 PM	Microsoft Word Doc...	25 KB
Map for Class Location and Parking	1/25/2018 3:11 PM	PNG File	144 KB

Email Subject Line: Orientation Survey Confirmation

Insert Date

Greetings!

Thank you for completing the Pre-Orientation Survey! We are really excited that you are still interested in participating in the NEW Soul Study!

During orientation, you will: meet study staff; learn more details about the study; learn about participant expectations; be able to ask any questions; and officially sign the consent form to participate. Additionally, dinner will be served.

Please see the attached document that provides directions to the orientation location and parking instructions. We look forward to seeing you soon!

If you have any questions, please feel free to contact Mary Wilson, Project Manager at 803.777.1902 or mjwilson@ mailbox.sc.edu.

Thank you!

(insert name)

Orientation Session

All participants must attend an orientation session, where they will learn additional details about the study and complete the study consent form.

Cohort 1:

- When:
  March 26, 2018 5:30pm- 7:30pm
  March 28, 2018 6:30pm- 8:30pm
  April 2, 2018 6:30pm- 8:30pm
  April 4, 2018 5:30pm- 7:30pm
  April 9, 2018 5:30pm- 7:30pm (available for back up)
  April 12, 2018 6:30pm- 8:30pm (available for back up)

Cohort 2: TBD

Orientation Location: Where: Discovery Building (915 Greene St, Columbia, SC 29201), room 140 and then the computer lab on the 4th floor in room 431 for ASA-24s and Baseline 1.

Items/Activities for orientation

- Attendance sheet
- Information packets with participant IDs, ASA-24 info, Baseline 1 info, study consent form, photo/video consent form, participant contact form, instructions for lab appointments, instructions for accelerometer use
- First dietary recalls completed in the computer lab
- Baseline 1 questionnaire completed in the computer lab
- Sign-up genius for baseline lab appointment completed in the computer lab
- Accelerometers to hand out for physical activity tracking periods
  - **NOTE**: See Accelerometer Manual of Procedures and inventory list on Dropbox : Soul Veg Study->Data->Accelerometers-> Accelerometer MOP
- Sheet to track accelerometers
- Nametags

Note: All documents can be found on dropbox.

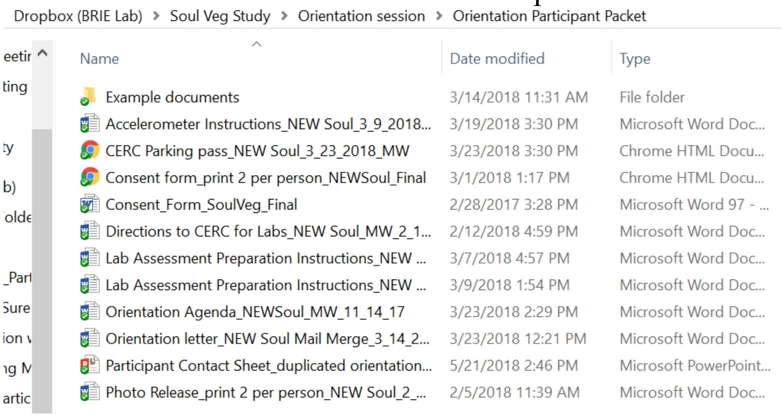

Prior to orientation all participants should have a participant ID number and corresponding ASA-24 login assigned to them so that on the day of orientation each individual can get this information in their orientation packet. Orientation will be held at the Discovery Building in room 140 and then later at the computer lab on the 4th floor in room 431. On the day of orientation someone from the study team should stand in the lobby to greet participants and direct them to the Discovery room 140. Additionally, posted signs for the NEW Soul study should be placed on the side doors in the lobby.

As each participant walks into room 140 someone from the study team should have a printed sign-in sheet with everyone's name on it. Ask the individual to identify his/herself. Once they do identify themselves, the names should be checked off and their information packet should be handed to them. Once they receive their registration materials, participants should be directed to the demonstration kitchen in Discovery room 101; it is directly across the hall from room 140.

Protocol for Unregistered Persons Attending Orientation

-Identify their name on the tracking sheet in the NEW Soul google drive (file name: Tracking)

-Assign them a Participant ID from the NEW Soul google drive (file name: Final_Usernames_And_Passwords_For_NSoul_2017_10_22_15_06_29.csv)

-Get one of the extra ready made orientation folders. Add their name, participant ID, username and password to the orientation letter (left hand side of the packet).

-Assign them an Accelerometer from the pack of extra accelerometers at the desk and add that information to the accelerometer tracking form on the NEW Soul google drive (file name: Updated_Accelerometer Inventory_NEW Soul_3_27_2018_MW.xlsx)

-In a sharpie marker, add a label to the accelerometer that you assign the person, and put their participant id on the sharpie. Then cover the new label with tape.

Orientation Agenda & Notes for Study Personnel

I. Welcome/Introductions/Overview of NEW Soul Study (30mins)
   a. Welcome
      i. Recruitment Video (Explanation of the benefits of losing a modest amount of weight (10-15 lbs)
      ii. Overview of Study and Participant Commitments and Intervention Activities
         1. Give schedule
         2. Ask to commit to completing makeups for any missed classes
         3. Staff Introductions
      iii. Overview of Scientific Portion of the Trial (I.e. control condition, random assignment, and attribution bias)
      iv. Small Group Activity (3-4) (pg 442 of Innovative techniques to address retention in a behavioral weight-loss trial)
         1. Two Pros and Two Cons of Being Randomly Assigned to Diet Group (PI leaves room for this portion)
         2. Groups Present
         3. PI led discussion and open-ended questions and reflective listening
         4. PI reiterates consideration of all pros and cons and commitment to self and the study prior to providing consent

II. Study Consent (10mins)

III. Computer Lab (60mins)
   a. ASA-24 Recall
   b. Baseline 1 Survey
   c. Signup for Lab and Baseline 2 Appointment
   d. Accelerometer Distribution

After registration and the serving of the food, Dr. Brie Turner-McGrievy will welcome participants, introduce the NEW Soul Team, provide a detailed overview of the NEW Soul Study and expectations and benefits of participating, and show a video of client testimonials. Immediately after, everyone will be asked to open up their packets and read over the study consent form and sign them. Participants will receive 2 copies. They will sign and date both and can keep one and turn one in. Everyone will then be asked to take out the photo/video consent form, read over it, and then sign. If someone decides not to participate at this time, they will be asked to stay to complete additional items in the computer lab. After the forms are signed, someone from the study team should collect them, mark that they were turned back in on the sign-in sheet and store them in an envelope. Dr. Turner-McGrievy will discuss the steps in the study and provide an overview of what participants will be completing in the computer lab.

Next, participants will be escorted upstairs by a member of the NEW Soul Team to the computer lab on the 4th floor in room 431. Everyone will be instructed to login into the ASA-24 website with their info. Some people may have difficulty with inputting information when it's their first time using the ASA-24. Study personnel should walk around the room and help individuals with their recalls. Once ASA-24s are completed, participants will be provided with a link to complete the Baseline Survey.

The Baseline Survey includes:

- Demographic information
- Current medications
- U.S. Adult Food Security Survey Module[18]
- Self-Efficacy for Diet Behaviors[19]
- Self-Efficacy for Green Eating Scale[20]

Note: Link to Baseline Survey- http://www.surveygizmo.com/s3/3911474/NEW-Soul-Study-Baseline-Survey

The baseline surveys will all be administered through survey gizmo. Participants will be directed to the website that should be already pulled up on their computer by staff ahead of time. Study personnel should continue to walk around the room to ensure that everyone is on the correct webpage. After a participant has completed all the surveys someone from the study team will work with them to sign up for their Baseline Lab Assessment appointment.

For the Baseline Lab Assessment appointment, an online sign up service (e.g. signupgenius.com) will be used to schedule appointments. The Baseline Lab Assessment appointment will be offered between April 12th - May 4th for Cohort 1 in 30/45 minute increments. Participants will only need to sign up for one appointment slot. Cohort 2 dates will be held in June 2019.

The final task during orientation will be to distribute accelerometers to each participant. Participants will be asked to wear accelerometers for the next 10 days and then return them during the baseline lab assessment appointment. The accelerometers are worn on the waist like a belt and should be worn at all times except during the shower or water activities (see the physical activity procedures section for details). A representative from the study team will hand out an accelerometer and will write down the accelerometer number next the study participant's ID on the tracking sheet.

INTERVENTION ACTIVITIES

Guidelines for Interacting with Participants

- **Always greet participants by name and with a smile. Address participants formally by Mr./Ms. and their last name.** Participants should be treated with the same respect and acknowledgment as a "customer".
    - If a participant arrives late to orientation or class meeting, the participant should be acknowledged by the facilitator with a smile and nod. Either openly or discreetly, welcome the participant and inform him/her of where you are on the agenda.
- **Maintain a pleasant and friendly attitude in all conversations with participants to place them at ease.** This positive rapport with the participant will ensure the willingness of the participant to remain in the study.
- **Assure participants' confidentiality.** Confidentiality should be maintained throughout the study. Anytime a participant asks about confidentiality reassure him/her that data and information will be de-identified, meaning their names won't be used for during data analysis and any document with one's name (i.e. consent forms) will be stored in a locked cabinet that only the research team will have access to. Other aspects of confidentiality to consider are not discussing any names or information outside of the study setting, making sure that written surveys are collected responsibly and not left in sight for anyone else to see them, and not discussing participants' medical records with anyone.
- **Be respectful to study participants**. This includes being punctual to study participants' classes, lab visits and measurements. To avoid unexpected delays, it is recommended that study personnel should arrive at the meeting locations at least 15 minutes prior to the appointment time. During measurement sessions, study staff should turn off personal cell phones. Study personnel should also maintain professional appearance, dress and speech. Specifically for dress, jeans are acceptable as long as they don't have holes or slits. No tank tops, open-toed shoes or clothes exposing midriff. Ladies, please refrain from wearing short skirts or dresses. Additionally, we are working with overweight or obese African American participants and study personnel should be sensitive and respectful about culture and issues related to body weight and image. Jokes or sarcasm relating to African American culture, weight or body image should be avoided.
- **Intervention Activities (Orientation, Labs and Classes) are NO CELLPHONE ZONES.** This is out of respect for our participants. Personnel assuming supervisory roles or who have been designated to call participants who are absent or having a difficult time locating the meeting location are allowed to have their cell phone during intervention activities.
- **Do not communicate voluntarily, or if asked, what diet you are following.** We are not allowed to discuss our dietary preferences with participants. This is a nutrition intervention study and we do not want to influence participants in any way. If a participant inquires about your dietary preference, politely explain to them that because this is a research study, you are not allowed to disclose any details about your own diet.
- **All staff and volunteers must complete staff/volunteer orientation requirements. This can be found on dropbox.**

  › Dropbox (BRIE Lab) › Soul Veg Study › Personnel › Staff and Volunteer Orientation

Consultants

Partner Restaurants/Chefs, other consultants or any company providing services or products to NEW Soul participants must complete a supplier packet. This can be found on dropbox. Consultant invoices should also be stored here. Catering consults should be asked to submit a list of ingredients for each dish and label each dish.

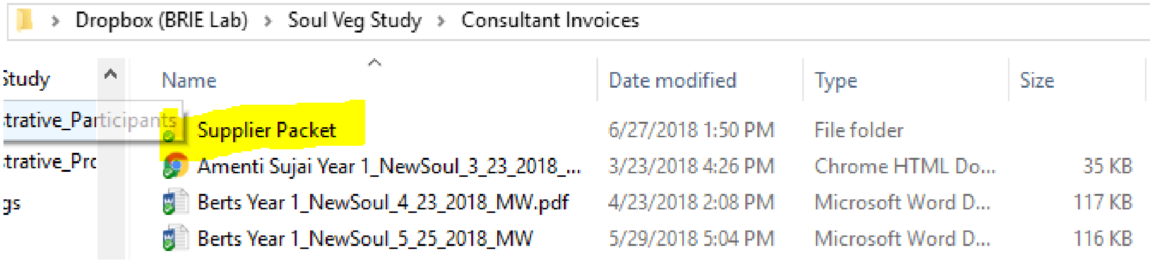

Overview of Classes

Classes will be held at the Discovery Building in the Cancer Prevention and Control Program's demonstration kitchen located on the first floor in the main lobby area in room 101. The classes are structured to be an hour and 15 minutes. Participants are required to attend weekly meetings for the first 6 months, bi-weekly meetings from months 7 to 12 and monthly meetings for months 13 to 24, with additional support delivered remotely after 6 months. Classes will be held in the evenings and participants will pre-register for a timeslot that works best for them. Each diet group will be offered a choice of two different meeting times on Mondays and Wednesdays, with the option to attend make-up sessions during the week or electronically. Classes will be led by an interventionist and other team members who are familiar with the diets. The basic structure of each class is a guided discussion and cooking demonstration for a particular meal. Each class will also have a "homework assignment," which will ask participants to make gradual changes for their particular diet assignment. For example, the vegan group will be asked to "eat vegan only for breakfast and lunch" after week 2. NEW Soul Curriculum can be found on dropbox.

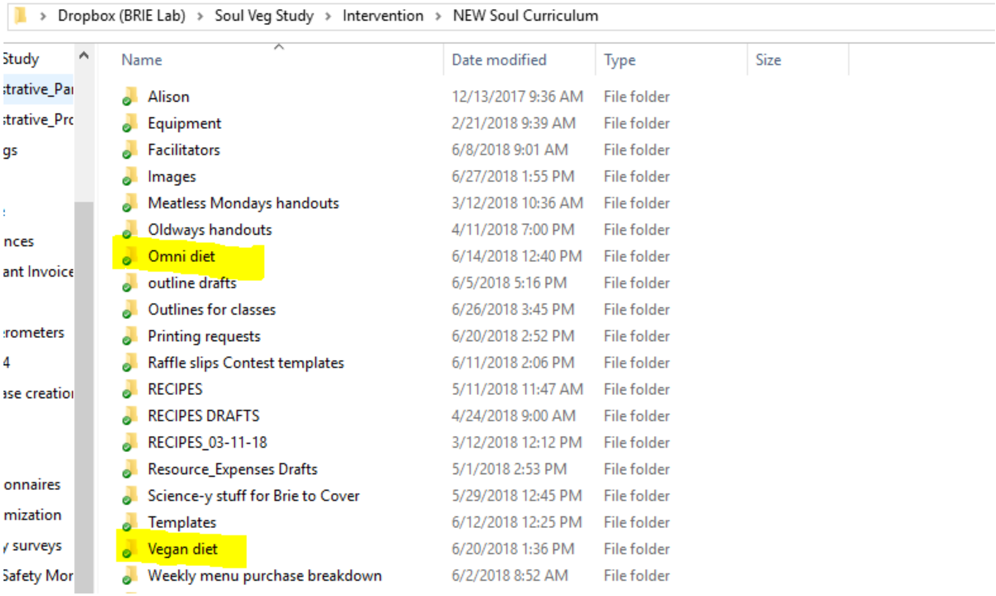

Notes from each class can be found on dropbox.

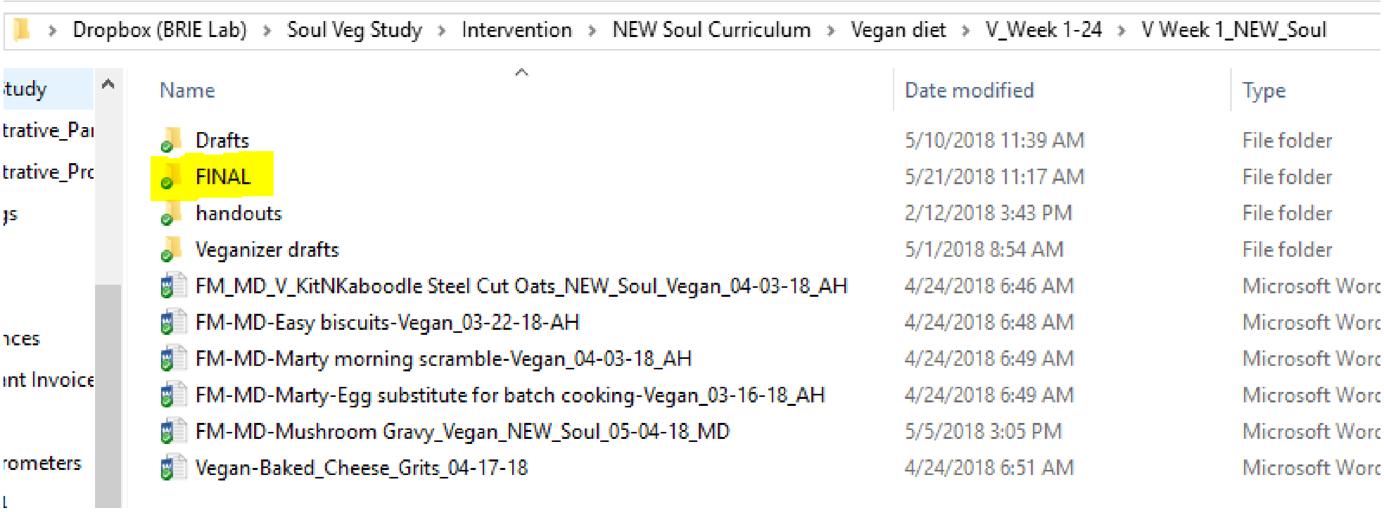

Procedures for Classes

Study personnel should have attendance sheets and have each participant sign in as they arrive. Participants that are bringing friends or family should also have the partner sign in. If there are upcoming lab assessments or baseline questionnaires to complete study personnel should remind participants to sign up for an appointment (which will be emailed to their email addresses).

CHECK-IN STAFF TASKS

CHECKLIST		NOTES
☐	Secure pre-packed check-in crate from Office 538 in Discovery	The crate should include an attendance list for all classes in the given week, participant and staff name tags, tabletop signs, table cloth, tablet and charger, camera, pens, additional dell laptop charger, and study cell phone. Also be sure to check the first drawer of the large filing cabinet that includes a cardboard box that is labeled "For Check-in". Also, the study cellphone may be plugged in under Mary's desk. Be sure to check that area.
☐	Retrieve table out of DISC 140 and place in hallway outside of the kitchen entrance.	
☐	Setup the table with the materials provided at least 30 minutes prior to the start of the first class. All name tags should be set out on the table for the associated class. Pull up the database and monitor incoming messages and reply as needed. Also monitor incoming calls to the study cellphone.	Sometimes people switch class days. If you notice that someone in the Vegan group who is normally in the Wednesday class and comes to the Monday class, retrieve their name tag from the Wednesday class envelop. Also make sure that when you collect their nametag, that the nametag is placed back in the Wednesday group envelop and not the Monday group. For your convenience, nametags have numbers on the back that correspond with the number on the envelop that it belongs in.
☐	Have participants put the date and initial by their name on the sign-in sheet. If they are in need of any handouts from missed classes, have them write which session they need on the sign-in sheet. Instruct them to then take a name-tag and direct them to the appropriate meeting location (140 or kitchen).	Have participants who arrive early for the 7:00pm class, sign-in and wait in 140 until the 5:30pm class is dismissed.<br><br>Note: There will be a raffle drawing on certain class days. When this applies, Marty will let you know. For days when drawings are being done, retrieve the participant ID number for each participant who attends, and place their ID number on a slip of paper and fold it up and place it in the appropriate class drawing container. Provider these containers to a kitchen assistant after the sign-in process is complete so that the drawing can be done at the end of class.
☐	If you need to validate parking for a participant. See the instructions below.<br><br>Website: https://innovista-park.com/Olv/Views/Login.aspx<br><br>Username (type in exactly with spaces :<br>Eating With Soul<br><br>Password: NEWSoulfb37*<br><br>Also, send Mary an email so that she can resend the participant his/her assigned parking pass.	- Login and click on the tab located in the top left hand corner that is labeled "eParcVal".<br>- Click into the box labeled "Ticket Number" that is located under the "Validate Ticket" heading.<br>- Follow the instructions in the image that pops up and enter the information exactly how it is requested.<br><br>PLEASE NOTE: When in event mode (occurs when there is an event at the Colonial Life Arena or Koger Center), no tickets can be validated. Participants will need to scan in and out of the garage with their limited parking pass or the image of their parking pass that they saved on their phone. If a participant does not have their parking pass, please contact Mary.

☐	Text all persons through the database (or call with the NEW Soul cell phone) who do not show up for class within 15minutes of class beginning. See template in the notes. The password to unlock the study phone is 201837.<br><br>Document responses as appropriate in the database. If they will not be coming to class, document that they will be absent. Additionally, if there is a response that they will not be attending class, please provide make-up options and document in database which make-up option they would like to sign up for. See notes.	Hi Mr./Ms._____, this is ____from NEW Soul. I wanted to check in with you to see if you were coming to class today. Thanks, ____<br><br>Vegan Group Make-up Options Specific for Cohort 1\ MONDAY CLASS<br>Thank you for your response, Mr./Ms. ____. We will miss you today, but you have two make-up options. We would love to see you on Wednesday for the vegan class at 7:00pm. If you are not able to make it on Wednesday, Mary will be sending out instructions on Thursday to access the makeup video that you can watch online. Please let me know which option will work best for you.<br>Thanks, ______<br><br>Omni Group Make-up Options Specific for Cohort 1 MONDAY Class<br>Thank you for your response, Mr./Ms. ____. We will miss you today, but you have two make-up options. We would love to see you on Wednesday for the omni class at 5:30pm. If you are not able to make it on Wednesday, Mary will be sending out instructions on Thursday to access the makeup video that you can watch online. Please let me know which option will work best for you.<br>Thanks, ______<br><br>Vegan and Omni Group Make-up Options for WEDNESDAY Class<br>Thank you for your response, Mr./Ms. ____. We will miss you today, but Mary will be sending out instructions on tomorrow to access the makeup video that you can watch online.<br>Thanks, ______
☐	Take pictures with the study phone as you are able. For guest chef cooking demos, provide the study phone to the facilitator to record a full video with the chef's permission.	Be sure to make an announcement that you are taking pictures in case anyone does not want to be in the picture.
☐	Retrieve envelop of nametags and completed in-class surveys from the facilitator once class is dismissed.	
☐	In between classes record attendance in database.	
☐	Prepare for next class and switch out name tags.	
☐	Retrieve envelop of nametags and completed in class-surveys from the facilitator once class is dismissed.	
☐	At the end of both classes, return all items to the cart, remove the table from the hallway by placing back in DISC 140.	Return documents (i.e. sign-in sheet) with identifying participant information to the locked drawer. Leave the cart in DISC 538 and place Mary's keys in an envelope and return to her mailbox located in the mail/copier room on the 5th floor.

KITCHEN CREW TASKS

-	Insert Day of the Week and # of Participants in each class<br>I.E. Cohort 1 Monday - V- 20 /Monday - O - 17
Meal service	Set out plates, utensils
	Serving spoons/forks
	Drinks
	Fold napkins
Recipe Demo	Prep herbs, additional ingredients
	Check demo ingredients for BOTH demos
Clean up	Put pre-class pots/dishes in dishwasher or hand wash
Class Tech	Get laptop, projector, screen from office
	Prep equipment - Check projector placement
Dining	***DEMO Service***
	Portion out meal components
	Replenish drinks/meal components
Clean up	***Between classes***
	Gather dishes/utensils
	Check Waters
Clean up	***End of DAY***
	All plates hand washed
	Dishwasher filled
	All cooking pans and utensils washed
	Projector/screen/laptop returned to office
	Sweep floor
	All napkins/rags into washer and turned on
	Ingredients labeled and refrigerated
	Extra handouts returned to office
	Counter tops wiped down
	Tables wiped down
	Dishwasher turned on
	Lights out

Weekly Emails/Posts
Each Thursday following classes, participants will either receive an email of the link to the weekly survey, class handouts, recipes, and announcements OR participants will be notified to access this information via the NEW Soul participant webpage.

Webpage- https://www.newsoul.org/participant/guest/login
Participant user name: Participant email address
Participant password: Participant ID #

Social Media Group and Audio Podcasts
In month 7, when classes go to every other week, participants will be offered a private online Facebook group that is moderated by study counselors to lead discussions to facilitate social support. Participants will receive audio podcasts and newsletters beginning in month 13. Participants will receive a notice via email to access this information via the NEW Soul participant webpage and will also be available via Facebook.

Participants without internet access (smartphone, home computer or library computer) will be mailed copies of the newsletters and podcasts on a CD. These participants will also be provided with the study counselor tips and posts that appear on Facebook via a handout.

Noncompliant Participants
Participants will have the opportunity to make up classes that they miss. The project manager will serve the role as primary retention facilitator. The study will also help ensure retention through raffles of small prizes at each meeting, as well as sending out birthday cards ("Dear, ____, We wanted to personally wish you a Happy Birthday. We hope that you enjoy it to the fullest!") to participants, additional cards as needed such as get well or condolenscens, and check-in calls from the project manager on a quarterly basis. As a best practice, core staff should sign cards and others as available. Participants will also receive phone calls/text message/email after each missed meeting (or missed podcast download) in order to ensure a make-up session can occur. All contacts with non-compliant participants will be tracked in the database.

Participant Expectations for Physical Activity
Participants will be asked to increase their walking as the primary way to improve physical activity (PA). Participants will be given the following exercise recommendations: strength training twice per week and ≥75 minutes of vigorous or ≥150 minutes of moderate PA per week.

Treatment Fidelity Checks
As outlined in the grant proposal, unannounced treatment fidelity checks will be conducted to ensure quality and consistency of intervention delivery. 15% of our classes will be rated on a quarterly basis (months 3,6,9,12,15,18,21). The project manager will arrange for these with a work study student or GA. The instructions and form can be found on dropbox.

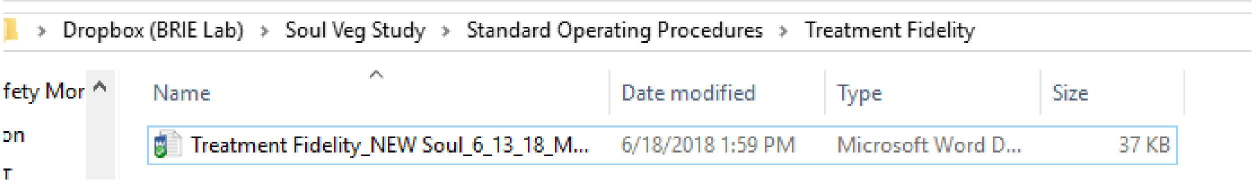

LAB ASSESSMENTS

Participant Instructions to Prepare for Lab Assessment Appointment (provide at orientation):

- Your lab assessment will include the following measurements: blood pressure (baseline, 6 months, 12 months, 24 months), height, weight, waist circumference, hip circumference, blood draw (baseline, 6, 12, and 24 months) and DEXA scan (baseline, 12 months, 24 months).

- You must fast! Please abstain from all food and liquid EXCEPT water for 12 hours before your scheduled appointment time.

    - Example: If your appointment time is at 6:00am, you should not have anything to eat (including candy) after 6:00pm on the previous day.

- Please drink plenty of water before your appointment though, as dehydration can make the draws very difficult!

- Please arrive to your appointment **on time** or 5 minutes early.

- Return your accelerometer during your scheduled appointment time.

LAB ASSESSMENT APPOINTMENT

DATE:________________________

TIME:_________________________

ADDRESS: 921 Assembly St., Columbia, SC 29208 (Room 315 on the 3rd floor; see CERC directions form)
Note: Building doors will be locked prior to 8:00am. Please call 803-777-4580 or 803-777-1902 for someone to let you in.

Lab Assessment Participant Reports
-After your 3 month assessment, you will receive a comparison report of your weight and diet changes (pulled from your dietary recalls) at baseline and 3 months.
-After your 6 month assessment, you will receive a comparison report of your weight, blood pressure, waist and hip circumference and diet changes at baseline, 3 months and 6 months.
-After your 12 month assessment, you will receive a comparison report of your weight, blood pressure, waist and hip circumference, diet changes, and DXA scan at baseline through 12 months. Note, the DXA scan will only be done at baseline, 12 months and 24 months.
-After your 24 month assessment, you will receive a comparison report on your weight, blood pressure, waist and hip circumference, diet changes, DXA scan and your blood draws from baseline through 24months. Please note, that all collected blood samples will all be ran at the same time at the end of the study or at 24 months.

Signing-up Participants for Lab Assessments for 6, 12, and 24 months

We will be using an online service (e.g. signupgenius.com) to have participants choose a time and date for their lab assessments. The available times for lab assessments will be from 6 am – 9:30am Monday – Friday over a 2-3 week period. Appointments will be in 30 minute increments, but note that the entire process is likely 30-40 minutes per participant. Study personnel should make an announcement in class a few weeks in advance. For participants that do not have internet access, study personnel should work with them to schedule a time and date during the classes at Discovery and have that time slot blocked out on the online scheduler. Lab assessments are conducted at the baseline 2 appointment, 6 months, 12 months, and 24 months.

Lab Assessment Reminders to Participants

Participants should receive a 3-day notice via email reminding them of their upcoming lab assessment. Participants should also receive a reminder text message reminder the day before their lab assessment. See the templates below.

Email Reminder

Good Afternoon Ms. __________,

As a reminder, your lab appointment is tomorrow from 7:30am-8:00am. Please review the instructions you received in your packet at orientation.

- **You must fast prior to your lab appointment!** Please abstain from all food and liquid EXCEPT water for 12 hours before your scheduled appointment time. **This includes coffee, tea, gum, mints, candy, juice, etc.**
  Example: If your appointment time is at 6:00am, you should not have anything to eat after 6:00pm on the previous day.
- Please **drink plenty of water** before your appointment though, as dehydration can make the draws very difficult!

- Attire: You may change into scrubs that we will provide once you arrive or you may wear a t-shirt and pants with no metal or zippers to your appointment. For ladies, underwire is ok.

Address: 921 Assembly St., Columbia, SC 29208
For any questions or concerns, please call 803-777-1902.
Thank you,
(insert name)

Text Message Reminder

Hello, Mr./Ms.____. As a reminder, your lab appointment is tomorrow from 8:00am-8:30am. Please review the instructions you received in your packet at orientation. Address: 921 Assembly St., Columbia, SC 29208. Please call (insert study phone) if you have any questions. Thanks, (insert name)

Operating Procedures for Lab Assessments (Baseline, 6 months, 12 months, 24 months)
Appointments

- When: Dates TBD
- Where: Public Health Research Center (921 Assembly Street Columbia, S.C. 29208), room 315 and 317

Items for lab appointment:

CERC will provide:

- Scrubs for them to change into upon arrival
- Clipboards
- Stadiometer for height
- Calibrated scale for weight
- Gulick for circumferences (can do these behind a screen if 316 is occupied)
- Desk space for blood pressure / administering questionnaires / handing out incentives, etc.
- DEXA scanner for body composition
- Everything for blood draws:
    - Alcohol wipes
    - Gauze / band-aids
    - Tourniquets
    - Biohazard disposal
    - Serum and EDTA tubes for collection
    - Needles / collection kits
    - Pipettes for transfer
    - Eppendorf storage tubes
    - Centrifuge
    - Ice (on the 4th floor)
- Staffing for blood draws and DEXA scan
- Storage cabinet with key

NEW Soul will provide:

- Sign-in sheets
- Accelerometers tracking sheet & laptop for downloading data
- Database report of incomplete assessments
- Lab assessment checklist
- Measurement tracking sheet for measurement coordinators
- Tablets/Laptops for picture taking and assessment completion
- Blood pressure machines and large cuffs & extra batteries
- Sample storage boxes and cooler to transport samples
- Granola bars (3g fiber/3g fat/6g sugar)*Must have vegan bars after baseline.
- Assorted juices
- Pens

- Parking Passes (from CERC)
- Stapler

Staffing Needs (minimum of 4)

- 1- Greeter/Checkout Coordinator
- 2- Measurement Coordinators
- 1- Floater/Backup Coordinator
  - o Direct participant as needed (i.e. to 315 from downstairs, to locker room, to room 317 for DEXA scan and blood draw)
  - o Open doors (doors are locked until 8 am)
  - o Wipe down all equipment to be used during the lab before any participants arrive and wipe down after each use.
  - o Fill in where needed (direction will be given by Lab Supervisor).
- 1- Lab Supervisor
  - o Place sign on rear entry door downstairs (near participant parking).
  - o Utilize the phone on the desk where blood pressures are being taken to call any participant who hasn't arrived within 10mins of their appointment time.
  - o Wait until last sample is processed and deliver to lab at school of medicine. While waiting on sample to be processed clean up and pack up supplies. Estimated leave time is10:30am.
  - o Assume roles of Floater/Backup Coordinator if there isn't one.

Project Manager Tasks for Lab Assessments

- All supplies will be at the lab upon arrival and labeled. Lab supervisor for the day should receive the key to the cabinet space to retrieve supplies.
- Lab times are expected to be from 6:00 am – 9:30:00 am each day specified. Participants will meet in room 317 to sign in with the study coordinator (the large exercise laboratory). **Study personnel should show up at least 15 minutes earlier than the first appointment scheduled.** The doors to the building might not be open until 8:00 am, and USC study personnel should be able to get in with their university ID by scanning it at the door.

The designated greeter for the day will track attendance and should go downstairs and look out for participants who are trying to enter the building and escort them upstairs. After the greeter signs in the participant, the greeter will ask the participant if he/she placed the parking pass in their front-left windshield. The greeter will take a photo of the participant (if the participant agrees to have this taken to track their visual progress) and then greeter will also have the participant return the accelerometer and document this on the accelerometer tracking sheet. The greeter will then, direct the participant to the measurement coordinator. The measurement coordinator will collect the participant's blood pressure, height, weight, and waist-to-hip circumferences in that order.

The measurement coordinator will document all results on the lab checklist and on the measurement coordinator tracking form. These documents can be found on dropbox in the “labs” folder for the specified lab assessment. See an example below of the forms used for baseline lab assessment.

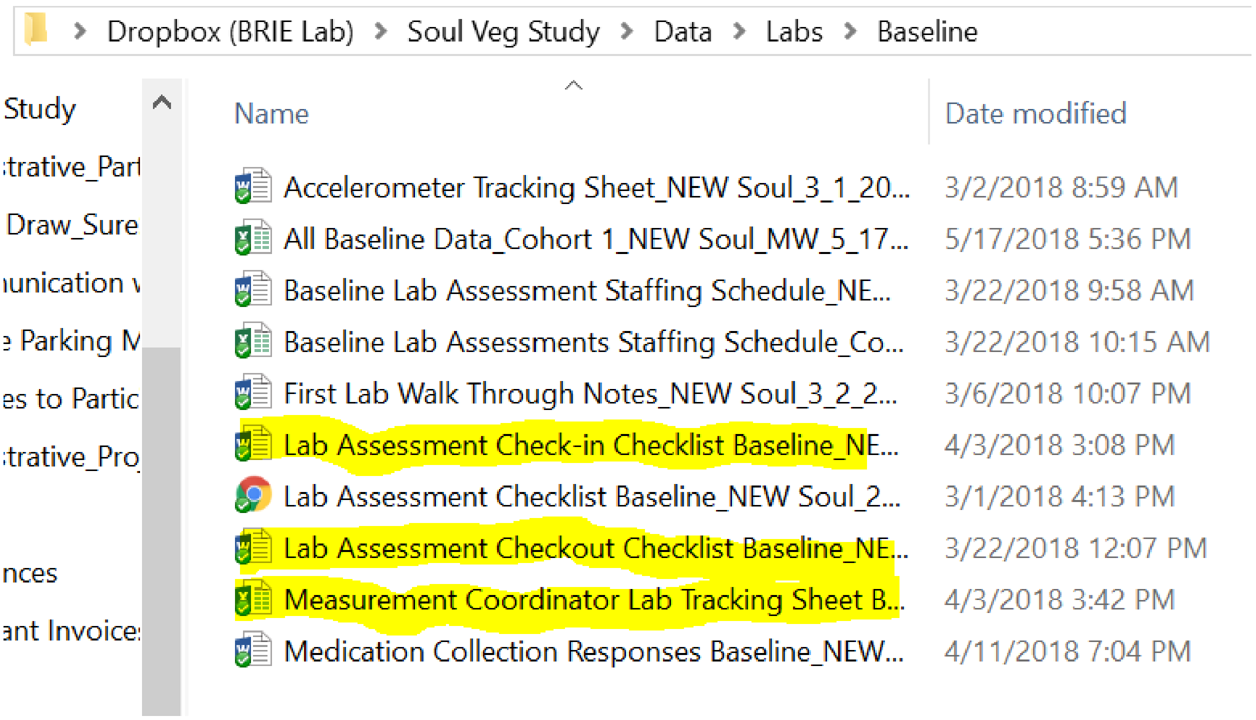

Note: Forms for each measurement time period will be in that particular folder. Baseline lab assessment forms will be found in the baseline folder. 3 month lab assessment forms will be found in the 3 month assessment folder.

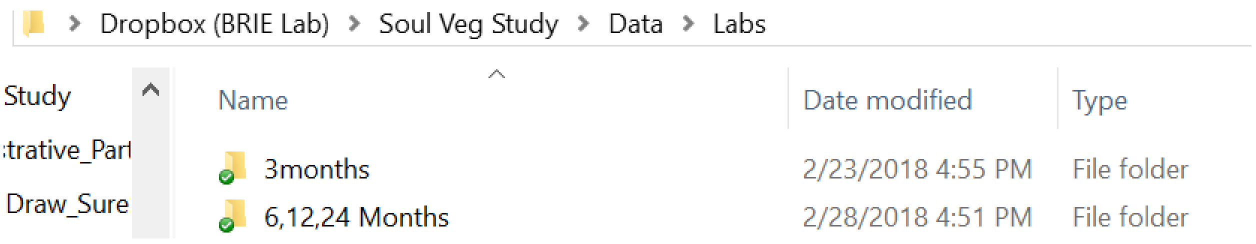

Procedures for Blood Pressure

Notes for Reference:

- Wear a watch if you have one or utilized the clock on the phone on the desk or the clock on the wall in CERC.
- BP should be taken in left arm. *Large cuffs are available when needed.
- Participant chair should be behind the desk facing the windows
- New 2017 ACC/AHA BP Guidelines

  Blood pressure categories in the new guideline are:
  - Normal: Less than 120/80 mm Hg;
  - Elevated: Systolic between 120-129 *and* diastolic less than 80;
  - Stage 1: Systolic between 130-139 *or* diastolic between 80-89;
  - Stage 2: Systolic at least 140 *or* diastolic at least 90 mm Hg;
  - Hypertensive crisis: Systolic over 180 and/or diastolic over 120

  Note: In the event of a Hypertensive crisis, the Lab Supervisor should be contacted. The Lab Supervisor will coordinate with CERC to get two manual BP readings. If the manual readings are below 180/20, participant can continue with the lab appointment. If the manual readings are above 180/20, the participant can not continue with the lab appointment and the Lab Supervisor and CERC Staff will further communicate with the participant.

Before taking a blood pressure measurement, instruct the participant to please do the following:

- Remove tight-fitting clothing from your left arm.
- Sit in the chair with your feet flat on the floor. Rest your left arm on the table so that the cuff is at the same level as your heart.
- Remain still and do not talk during the measurement.

1. Blood pressure will be assessed using an Omron Automatic Blood Pressure Monitor with Printer (Model HEM-705CP). Before taking each participant's blood pressure, hold down the "M" button and "start" button simultaneously for more than 2 seconds to clear the monitor of any previous readings.
2. After a 5-minute rest period, the blood pressure will be assessed.
3. Wait 2-3 minutes between measurements. The wait time allows the arteries to return to the condition prior to taking the blood pressure measurement.
4. A minimum of two readings will be taken, and the average of those readings will be used. If there is a >5 mmHg difference between the first and second readings, additional (one or two) readings will be obtained, and the average of these multiple readings will be used.[26]
5. Document all results on the lab checklist and on the measurement coordinator tracking form.
6. Print the monitor receipt by pushing the "all data" button and staple to the participant's lab checklist.
7. Remember to clear the monitor before taking the next participant's blood pressure.

Procedures for Height

The first step is to calibrate the stadiometer (height measuring scale). This is done by using a level to determine if the measuring arm is held at a 90° angle to the measuring scale. The second step is to have the subject change into the scrub apparel that is provided (will use in weight measurement, immediately following).

Procedures for taking height:

1. The subject will remove his/her shoes and socks.
2. The subject will stand directly under the stadiometer with their heels together, an inch from the wall (or on the blue tape on the floor), and knees facing forward.
3. Their back and buttocks should touch the wall.
4. The subject should stand as straight as possible and look directly straight ahead.
5. The measuring arm should compress as much hair as possible and remain at a right angle to the measuring scale.
6. Participant should duck from underneath the stadiometer so that the measurement can be noted.
7. Measurements should be recorded to the nearest 1/10 cm.
8. 2 measurements should be taken. The measurements should be within 4mm of one another. Otherwise, a third measurement will be taken.
9. If the subject's height exceeds the measuring capabilities of the stadiometer, height will be measure by utilizing a tape measure.
10. Document all results on the lab checklist and on the measurement coordinator tracking form.

Procedures for Weight

The first step is to calibrate the weight scale. This is accomplished by placing designated calibration weights on the scale and adjusting the scale to the proper pre-determined weight. The second step is to have the subject change into the scrub apparel that is provided (if not already done for height measurement).

Procedures for taking weight:

1. The subject will remove his/her shoes and socks.
2. The subject will stand motionless on the weight scale (facing the exit sign in room 316) until a final measurement is obtained by the Energy Balance staff.
3. Wait until the scale beeps before recording the measurement.
4. Measurements should be recorded to the nearest 1/10 kg. Two measurements should be made and be within 0.1kg of one another. Otherwise, a third measurement should be taken.
5. Document all results on the lab checklist and on the measurement coordinator tracking form.

Procedures for BMI (will not be taken during lab)

BMI is obtained by measuring the subject's height and weight, if necessary convert to kilograms and meters. Then substitute the information into the following equation:

$$\text{BMI} = \text{body weight in kg} / \text{height in meters}^2$$

For example:

Body weight – 86.4 kg

Height –183 cm (convert to meters by dividing by 100) = 1.83 m

BMI – $86.4 / (1.83)^2 = 25.8$

Procedures for Waist Circumference

Notes for reference:

- Measuring tape for hip and waist circumference in far drawer closest to the window/scale.
- When conducting this measurement, it should be male-to-male or female-to-female. No male should measure the waist circumference of a female and vice versa. See Lab Supervisor to coordinate when necessary.

The first step is to calibrate the tape measure. This will be accomplished by measuring a pre-determined object for accuracy of the tape measure. A spring-loaded tape measure will be used on all waist circumference measurements.

Procedures for measuring waist circumference:

1. Measurements should be made with a spring-loaded, flexible but inelastic tape measure
2. The tape measure should be placed directly on the skin surface (no clothing on the area) without compressing the subcutaneous adipose tissue.
3. The tape should be pulled only until to the point where the entire red ball is just visible.
4. The subject should be standing, arms extended at a 90° angle to the floor, feet together, eyes forward focusing on a spot on the wall, abdomen relaxed (at the end of normal expiration), and with a normal breathing pattern.
5. The measurement should be taken horizontally 2 inches above the umbilicus.[24]
6. Measurements should be recorded to the nearest 1/10 cm.
7. 2 measurements will be taken. Measurements should be within 4mm of one another. Otherwise, a third measurement will be taken.
8. Document all results on the lab checklist and on the measurement coordinator tracking form.

Procedures for Hip Circumference

Notes for reference:

- Measuring tape for hip and waist circumference in far drawer closest to the window/scale.
- When conducting this measurement, it should be male-to-male or female-to-female. No male should measure the waist circumference of a female and vice versa. See Lab Supervisor to coordinate when necessary.

To measure hip circumference (measured against clothing):

1. Have the participant turn to the side.
2. Make the measurement at the maximum protuberance of the buttocks.[25], making sure the tape is level.
3. Measurements should be recorded to the nearest 1/10 cm.
4. Measure the hips twice. If the numbers are different by 2-3 inches, take the measurement a third time.
5. Document all results on the lab checklist and on the measurement coordinator tracking form.

Managing multiple participants

Sometimes two or more participants may show up around the same time. The study coordinator should prioritize assisting participants based on the scheduled appointments. For example, if participant A is signed up for a 9:00am appointment and participant B is signed up for a 9:30am appointment, but participant B also arrives at 9:00am, participant A should be assisted first. Study personnel should explain to participants who arrive early that efforts will be made to accommodate them and that they will be assisted at the study personnel's earliest convenience. To accommodate participants, study personnel may have the early arrival do their DEXA scan prior to their measurements being taken. Study personnel should not send participants to get blood drawn prior to having their blood pressure taken, as this could potentially have a negative effect on their blood pressure reading. Another option is to have early arrivals see the check-out personnel to complete any surveys or ASA-24s that's needed while they wait.

Dressing for DEXA scan

Because the DEXA scan is using x-ray technology to scan one's bone density, participants need to be dressed appropriately before getting scanned. You will receive scrubs to change in to at the lab. All jewelry should be taken off prior to the DEXA. There are keys and locks in the exercise lab that study personnel can give participants to use to lock up and secure possessions. The room for the DEXA is down the end of hallway on the third floor (to the left upon exiting the exercise lab).

***In an effort to minimize time for changing, participants may be instructed beforehand to wear a t-shirt and pants that do not contain any metal buckles or zippers.

NOTE: Underwire is ok. Female participants SHOULD NOT be instructed to remove their bra.

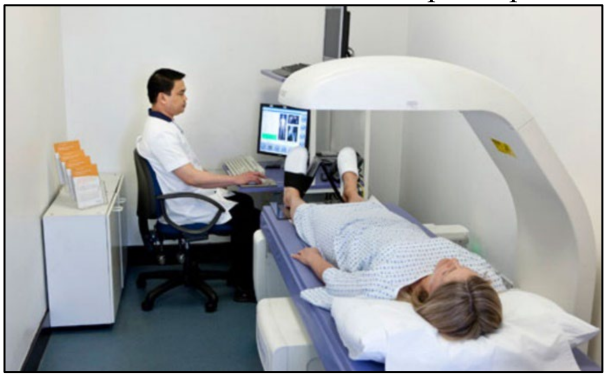

Example of DEXA Scan

Procedures After Collection of Measurements

After collecting these measurements, the participant will then be directed to the CERC staff.

The CERC staff will be doing measurements, which will be: DEXA scan and blood draw. CERC staff will distribute the nutritious snack. After the participant has completed all of his/her measurements, they will see the designated study personnel to check-out.

Procedures for Check-out

The check-out process as outlined on the participant check-out list, will include the participant changing back into their clothes, the check-in/check-out coordinator verifying that all measurements have been completed and that notes are indicated for incomplete measurements. This also includes the accelerometer assistant verifying that data was collected and downloaded. The check-in/check-out coordinator will also verify that all questionnaires have been completed. If not, the check-in/check-out coordinator will provide the participant with a computer to complete onsite. The check-in/check-out coordinator will also verify that the medication collection responses are valid and will update the form with any changes to medications if communicated by the participant. After the last participant leaves, study personnel will return the blood pressure monitors, attendance and tracking sheets and accelerometers to the specified secure storage site (locked cabinet in 317). The key for the cabinet should be taped securely underneath the desk located immediately to the left of the cabinet space.

The designated lab supervisor of the day will deliver participant blood samples to Dr. Angela Murphy at the USC School of Medicine. On the day of pickup/delivery of samples, also included should be an inventory form of all samples (total number of samples included and list of participant ids that are representative of the samples). The address is 6439 Garner's Ferry Rd. Her lab is in building 2, room B16. It is okay to park in any available spot.

For any questions, concerns, or emergencies when delivering samples, Dr. Angela Murphy can be reached at 803.360.1362. Also, Alex (her Ph.D. student) can be reached at 217.898.3298.

Verifying Lab Measurements (Baseline, 3 months, 6 months, 12 months, 24 months)

Identified staff will remain after the last participant leaves to verify that measurements recorded on the measurement coordinator tracking form and the check-in form match. One person will then enter the measurements in the database. The Project Manager will verify the entered measurements by comparing what was entered in the database to the check-in form and measurement coordinator tracking form. An electronic form should be updated to track blood sample collection for each participant at each lab point.

Procedures for Randomization of Diet Groups After Baseline Assessments

Participants will be randomized once all baseline measurements have been completed.

NEW Soul randomization procedures:

1. Brent Hutto will create a random list of 1's (vegan: Intervention) and 0's (omni: control) in blocks of 10. There will be separate blocks for women and men such that there will be 8 blocks of 10 for women (so 80 spots total) and 2 blocks of 10 for men (so 20 spots total).
2. As participants complete the lab assessment and have done all baseline measures, that day, someone who is blinded to participant identity will add them (add ID #) to the next spot on the blocks of 10 (the male or female blocks).
3. Participants will not be told what group they are in but instead will be told their choice of meeting times and days (For example, 1/Vegan, then Mon 5:30pm or Wed 7pm; If 0/Omni, then Mon 7pm or Wed 5:30pm).
    a. Participants will be contacted the day they are randomized and asked to select which time/date they plan on attending the first class.

Procedures for Distributing Incentives at 3, 6, 12, and 24 Months

Participants will be provided $10 for completion of the 3-month assessments, $50 for completion of the 6-month assessments, $50 for the 12-month assessments and $100 for the 24-month assessments. Incentives will be distributed during the correlating lab visits.
Check-out personnel will follow all check-out instructions as outline in the baseline lab assessments._After the participant has completed all of his/her measures, and surveys if necessary, the study coordinator or volunteer managing the participant check-ins that day should give the participant the incentive which will be in a sealed envelope. Ask the participant to open the envelope and then have them sign and date their name on a sheet which will be provided to the study coordinator. It is essential to get each participants' signature so that there is verification that he/she received the incentive. Any individuals that do not show up to their appointments can be accounted for when they reschedule.

Procedures for Lab Assessments at 3 months

Lab assessments will be conducted in conjunction with the normally scheduled weekly classes. Round 1 of assessments will happen during the specified week, and Round 2 of assessments will occur during the following week. 3 month assessments will include 3 dietary recalls that will be sent out 2 weeks prior to the scheduled assessment along with the 3 month survey. Before class, blinded assessment staff will record the weight of participants.

Cohort 1: Round 1- 7/23/18 & 7/25/18
Round 2- 7/30/18 & 8/1/18

Cohort 2- TBD

Procedures for Serum Preparations

1. Collect two vacutainers of whole blood in serum tubes (red-topped tubes).

2. After collection, allow blood to clot undisturbed at room temperature and away from direct sunlight for 30 minutes.

3. Remove the clot by centrifuging at 1,000–2,000 x g for 10 minutes.

4. Label 6 Eppendorf tubes for storage. Information should be written **directly on the tube** with a fine tip sharpie. Each Eppendorf should include the ID, date and time of collection, and the type of sample (i.e. **S** for serum) and measurement period (i.e. BL for baseline, 6 for 6 months, 12 for 12 months, and 24 for 24 months).

5. Immediately after centrifugation, aliquot the liquid component (serum) into Eppendorf tubes (0.5-1.0mL of serum/Eppendorf tube).

6. While handling samples, maintain serum samples at 2-8°C.

7. Following processing of serum, NEW Soul staff will deliver in sample storage boxes (on ice) to Dr. Angela Murphy's lab (Building 2, room 16 SOM).
    a. Sample storage boxes are color coded based on the measurement period. Blue=baseline; Pink = 6 months; Orange = 12 months; Yellow = 24 months

Procedures for Plasma Preparations

1. Collect two vacutainers of whole blood into EDTA-treated tubes.

2. To remove cells from plasma, centrifuge tubes for 10 minutes at 1,000-2,000 x g.

3. Label 6 Eppendorf tubes for storage. Information should be written directly on the tube with a fine tip sharpie. Each Eppendorf should include the ID, date and time of collection, and the type of sample (i.e. P for Plasma) and measurement period (i.e. BL for baseline, 6 for 6 months, 12 for 12 months, and 24 for 24 months).

4. Immediately after centrifugation, aliquot the liquid component (plasma) into Eppendorf tubes (0.5mL of plasma/Eppendorf tube).

5. While handling, maintain plasma samples at 2-8°C.

6. Following processing of serum, NEW Soul Staff will deliver in sample storage boxes (on ice) to Dr. Angela Murphy's lab (Building 2, room 16 SOM).
   a. Sample storage boxes are color coded based on the measurement period.
      i. Blue=baseline; Pink = 6 months; Orange = 12 months; Yellow = 24 months

Procedures for Participants Whose Samples Can't be Collected by CERC

In the event that CERC is not able to obtain a blood sample from the participant, CERC staff will inform the participant that the Project Manager will make contact with the participant to visit Sure Screen Labs, LLC to have the sample drawn at no charge to them. The Project Manager should call first and follow up with an email. See email template below.

Hello Mr./Mrs.

NEW Soul has secured a contract with Sure Screen Labs (a local arm of Labcorp) to collect your blood sample. They are located at 2015 Assembly St., Columbia, SC 29201. Their phone number is 803-256-9535.

1) **Please call them at your earliest convenience** and schedule an appointment **(WE WOULD PREFER AN APPOINTMENT THAT IS MORNING OR EARLY AFTERNOON)** to get a fasting blood draw done by (insert date). With our contract you only have to show up and they will invoice us for payment. **Please plan for approximately <u>15 minutes</u> for your appointment.**
2) **Email, text or call me to inform me of your appointment day and time.**
3) **When you go to your appointment, please take a completed copy of the attached appointment form.**

Please drink at least 2-3 bottles of water prior to your appointment time that morning to make sure that you are well hydrated. Additionally, remember **<u>You must fast prior to your lab appointment!</u>** Please abstain from all food and liquid EXCEPT water for 12 hours before your scheduled appointment time. **This includes coffee, tea, gum, mints, candy, juice, etc.** Example: If your appointment time is at 6:00am, you should not have anything to eat after 6:00pm on the previous day.

Kindest Regards,
(Insert Name)

NOTE: Appointment form for Sure Screen Labs can be found on dropbox.

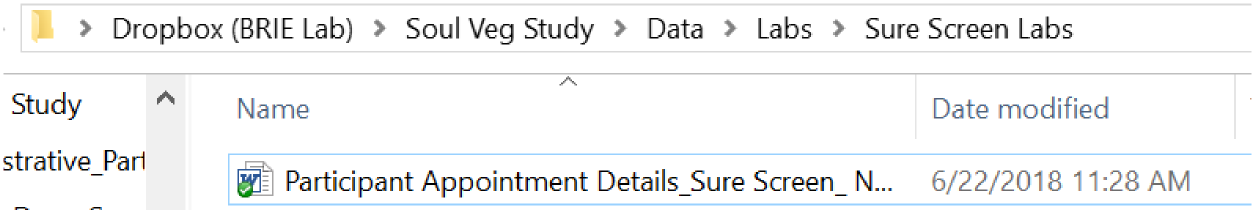

MEASUREMENTS

Summary of All Measures Being Collected

**Study Year**	**Year 1** 5/1/2018-4/30/2019				**Year 2** 5/1/2019-4/30/2020			
**Calendar year**	2018				2019			
**Measure**	0	3	6	12	15	18	21	24
Demographic Information	X							
U.S. Adult Food Security Survey Module[18]	X	X	X	X				X
Height (baseline only) and Weight	X	X	X	X				X
Self-Efficacy for Diet Behaviors[19] and Self-Efficacy for Green Eating Scale[20]	X	X	X	X				X
Dietary Intake (ASA-24)	X	X	X	X				X
Physical Activity (ActiGraph Accelerometer GT1M model)	X		X	X				X
Blood Pressure	X		X	X				X
Fasting Lipids, Glucose, and Insulin	X		X	X				X
Body Fat (DEXA Scan)	X			X				X
Waist-to-Hip Circumference	X		X	X				X
Current Medications	X	X	X	X	X	X	X	X
Dietary Acceptability[21-23]		X	X	X	X	X	X	X
Dietary Adherence (assessed Weekly and via diet recalls)	X	X	X	X	X	X	X	X
Attendance and Use of Intervention Components	X	X	X	X	X	X	X	X

COHORT 1

Baseline: April-May 2018	6months: November 2018	12 months: April 2019	24 Months: July-August 2020

COHORT 2

Baseline: June 2019	6months: November 2019	12 months: June- July 2020	24 Months: April-May 2021

Procedures for Dietary Recalls

Overview of ASA-24

The ASA-24 is the National Cancer Institute's automated system for tracking participants' dietary information. It asks participants to record all meals and beverages consumed in the preceding 24 hours at the time of doing the recall. For this study we want to focus only focus on the previous day. So, if a participant is doing a dietary recall on a Tuesday, they should only record all meals and drinks he/she had on that Monday.

Throughout this study participants will be asked to complete 3 unannounced recalls (2 week day recalls and 1 weekend recall. Participants will complete recalls in person at the orientation and at two other points in time that are listed below based on assigned class days. The goal is to have a day in between sending out emails to request recalls. Recalls can be tracked as shown in the ASA24 Recall Tracking file found on dropbox.

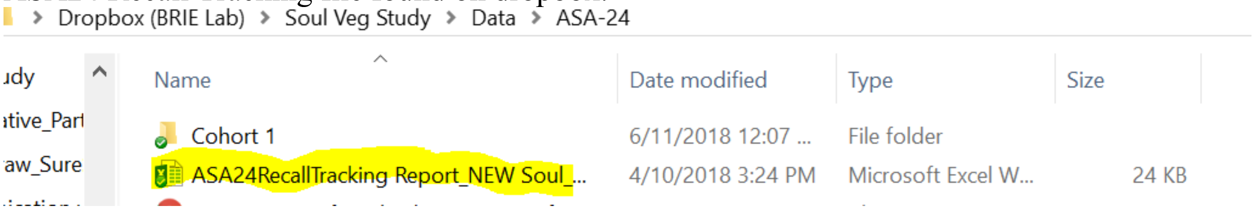

<u>Monday Class</u>
1st Recall (Weekend)- Day of Orientation
2nd Recall (Weekday)
3rd Recall (Weekday)

<u>Wednesday Class</u>
1st Recall (Weekday)- Day of Orientation
2nd Recall (Weekday)
3rd Recall (Weekend)

After that point, they will be asked to complete them at home or at their lab appointments at 3 months, 6 months, 12 months, and 24 months. An email with the link to the ASA-24 website will be sent to participants or a dietitian will call participants and do it by phone if they do not have internet access. Below are steps for sending and tracking recalls:

E-mail Link to Complete ASA-24

1st Notice

Between 8:00am and 9:00am, send both an email and text message to the intended participant with a link to the website and ask them to complete it before the end of the day. It is recommended you include their username and password in the first few emails in case they forget their login info. Below is the email template.

Dear (participant name),

We are asking you to complete a dietary recall today. Please log in to the website with your user name and password to complete it:

- Link to Food Recall: https://asa24.nci.nih.gov/

- Use your assigned user name and password (note that it is case sensitive):
    - User name: DTime184
    - Password: Hpeb!802

Please note that you must complete the dietary recall in one sitting or all at one time. This means that you are not able to complete half of it and login back in to complete the rest at a later time.

Thank you for your continued participation in the NEW Soul Study!

Warm Regards,
(insert name)

Below is the text message template.

Hi! We are asking you to complete a dietary recall today. Please log in to the website with your user name and password to complete it. Your username and password has been sent to your email. Remember that you once you start your recall you must finish it. The link to the food recall is https://asa24.nci.nih.gov/

2nd Notice

Between 12:00pm and 1:00pm, send a reminder email and text to the intended recipient with a link to the website and remind them to complete it before the end of the day. Below is the email template.

Dear (participant name),

If you have already completed your dietary recall, THANK YOU!
If you have not yet completed your dietary recall, please take a minute to do so. You must complete your recall before 11:59pm. Please log in to the website with your user name and password to complete it:

- Link to Food Recall: https://asa24.nci.nih.gov/

- Use your assigned user name and password (note that it is case sensitive):
    - User name: DTime184
    - Password: Hpeb!802

Please note that you must complete the dietary recall in one sitting or all at one time. This means that you are not able to complete half of it and login back in to complete the rest at a later time.

Thank you for your continued participation in the NEW Soul Study!

Warm Regards,
(insert name)

Below is the text message template.

Hi! If you have already completed your dietary recall, THANK YOU! If you have not yet completed your dietary recall, please take a minute to do so. Your username and password has been sent to your email. Remember that you once you start your recall you must finish it. The link to the food recall is below. https://asa24.nci.nih.gov/

3rd Notice (for participants who have not yet completed their recall)
Between 7:00pm and 8:00pm, send a last-call reminder text to the intended recipient with a link to the website and remind them to complete it before 11:59pm. You can logon to the asa24 website to view a report to see who has not yet completed their recall and send messages to only those individuals. *See tracking the recall on the next page. Below is the text message template.

Last Call for Dietary Recall- If you have not already completed your recall, please complete your dietary recall before going to bed. They must be submitted before 11:59pm. Your username and password has been sent to your email. The link to the food recall is below. Thanks so much!
https://asa24.nci.nih.gov/

Tracking the Recall

Log into the ASA-24 researcher website. Note that this is a separate website from the user website. The researcher website is: https://asa24.nci.nih.gov/researchersite/

Click track recall/record at the top of the page:

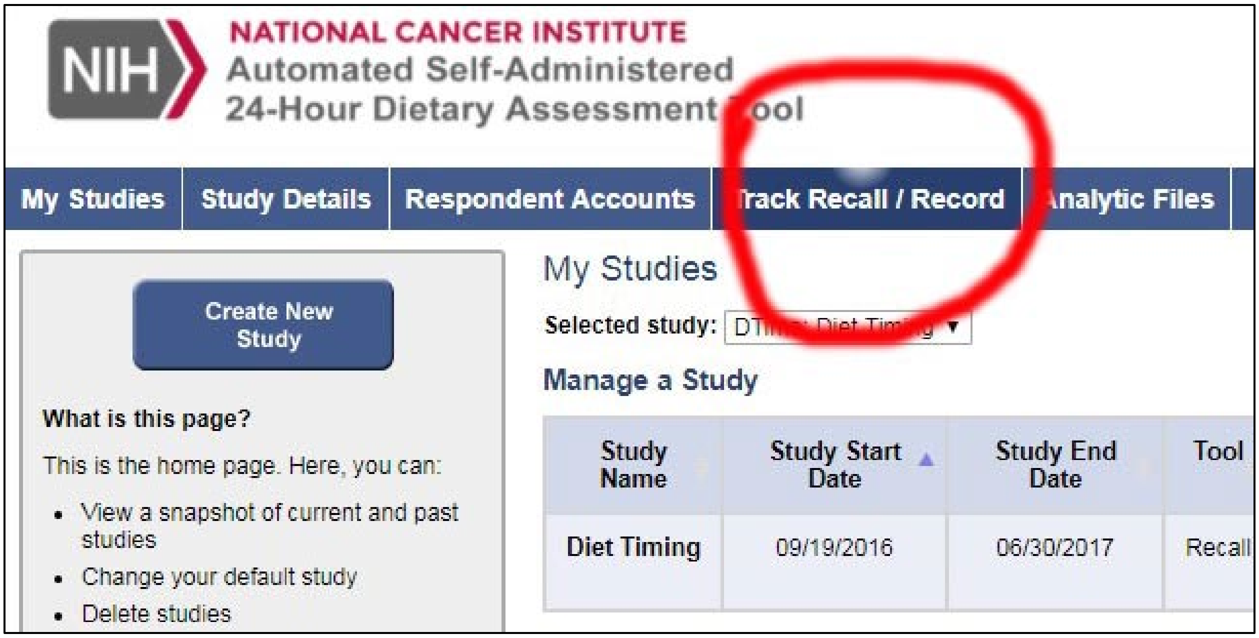

Click the recall/record button:

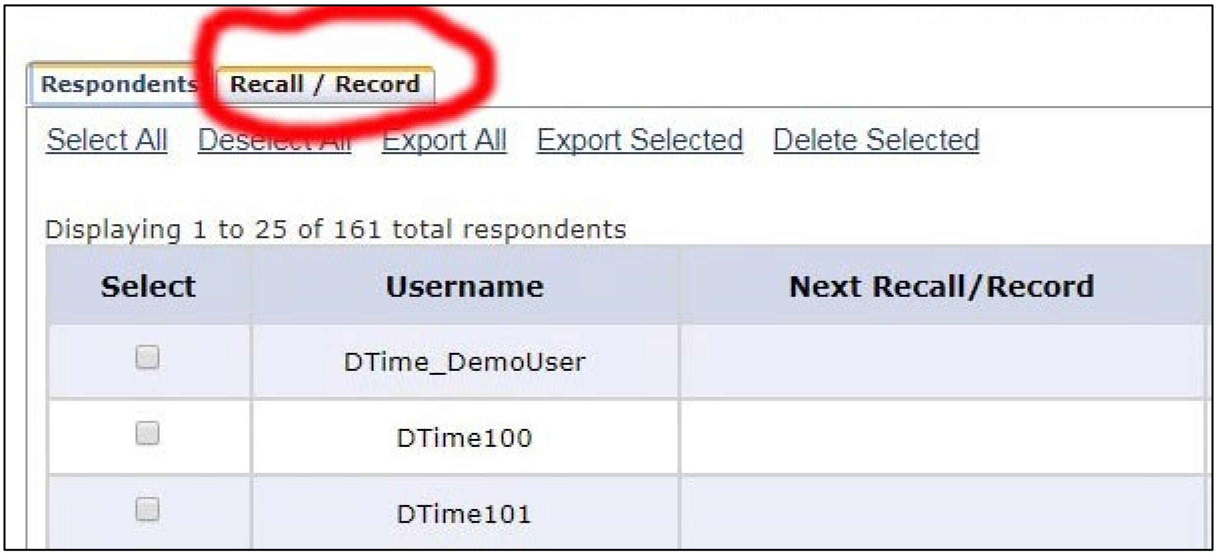

You will see each study participant's user name and the completion status of their recall. It should say "complete" and have the estimated number of calories. Note that it takes several hours for ASA-24 to process the information (~12 hours after the participant entered in all of

their info). You may have to wait till the next day to check that the recall information came through. If you see the status “quit” this means that the user either exited the recall without finishing it or they got booted out from the ASA-24.

Select	Username	Reporting Date	Completion Status	Number of Sessions	Total Session Duration	Language	Calories (kcal)	Number of Eating Occasions
☐	DTime115	9/26/2016	Complete	1	31	ENG	2471.760696	5
☐	DTime115	9/25/2016	Quit	1	37	ENG	628.2724	7
☐	DTime115	10/2/2016	Complete	1	51	ENG	1768.0905	7
☐	DTime146		Not Started	0	0		0	0

Incomplete Dietary Recalls

There will be times when participants do not complete their recalls. This may be due to several reasons, such as forgetting to do the recall, having technical difficulty with the ASA-24, or a loss of motivation. Usually a reminder email or a courtesy call will prompt the participant to complete it. If there is a repeated pattern of incomplete recalls it may be best to have a phone call with the participant and assess why they are not completing the recalls. You can gently remind him/her that they will receive incentives for completing all of the study’s surveys and questionnaires.

Common Technical Problems with the ASA-24

- *Logging in and out:* The ASA-24 can only be completed in one setting. Meaning that participants cannot enter in partial information and try to log in later to complete it. The ASA-24 will lock the user out from attempting to log in a second time in the 24 hour window. If this is the case, the participant will need to wait until the next day to log in and complete the information.

- *Server goes down:* There are unanticipated times when the ASA-24 can crash while the user is entering in his/her information for the night. Unfortunately the user will have to wait until the server is functioning again and re-enter their information.

Procedures for Accelerometers to Track Physical Activity

An ActiGraph accelerometer (GT1M model, ActiGraph, LLC, Fort Walton Beach, FL) will be used to objectively assess minutes per week of moderate- to vigorous-intensity PA. The accelerometer will be worn on the right hip for all waking hours, except when in water, for 7-10 consecutive days during each time point (baseline, 6 months, 12 months and 24 months). A 60-second epoch (time interval) will be used, and at least 5 days (including a weekend day) with at least 10 hours/day wear time will be required. Participants who do not meet wear-time criteria will be asked to wear the monitor a second time. Instances of consecutive zeroes for 60+ minutes will be removed from analysis because of presumed non-wear time. Counts/minute of ≥1,952 will be classified as moderate- to vigorous-intensity PA.

Participants will be wearing accelerometers for a 7–10 day period during each time point (baseline, 6 months, 12 months, and 24 months). Accelerometers should be fully charged before they are handed out to participants. Study personnel should record the participant's ID number next to the assigned accelerometer handed out to the individual on a sheet of paper. Accelerometers are to be worn around the waist like a belt and the device should be placed towards the right side of the individual's hip. Accelerometers are to be worn at all times except for showering or swimming because they are not water proof.

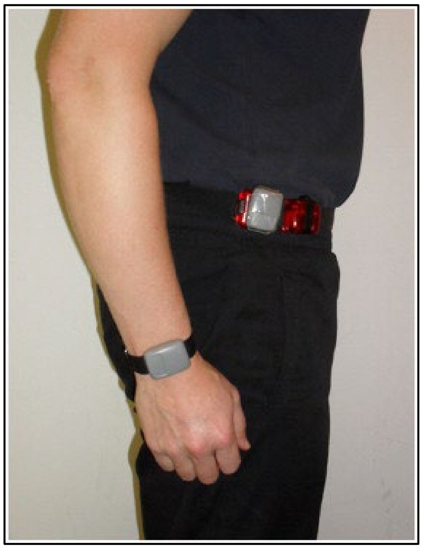

Example of accelerometer placement

DATA MANAGEMENT

Collection and Storage

Paper documents, like attendance sheets, need to be entered online and stored in a locked cabinet. This study will have two online storage clouds:Onedrive and Dropbox, and a secure online database. Dropbox tends to be preferred because it is easier to edit files, however Onedrive must be used for anything that has participants' names. Dropbox cannot have any identifiable information since files can be downloaded from an unsecure database. Instead we will use participant ID numbers for Dropbox. After the information has been entered online all paper documents will be stored in study's filing cabinet located in office 538.

Tracking Data

To maintain secured online records, all written data, such as attendance information, should be entered online in OneDrive (if there is identifying information). All written data without identifying information will be entered online in Dropbox. This study will also have an online tracking database- https://newsoul.org/staff/guest/login.php. Staff must see Project Manager to retrieve a user account and to get trained on using the system.

Study Grant Application

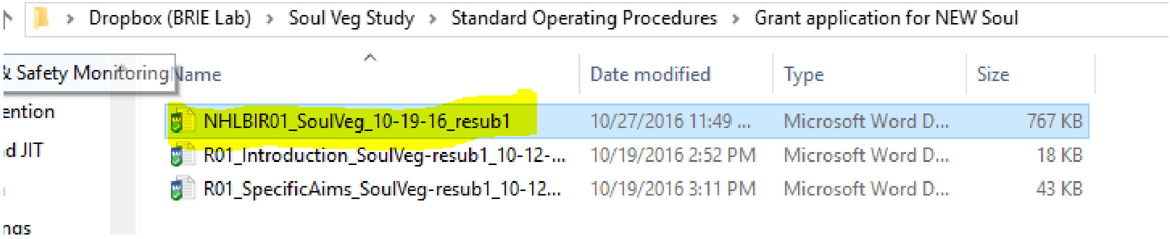

Study Related Accounts

Found on one drive.

Study Staff & Volunteers

Staff- All staff persons should be hired through Dantz Woodberry or Brittany Hammond.

Volunteers- All NON-STUDENT volunteers must review and sign U of SC's Volunteer Activity Form prior to their start date. The volunteer's immediate supervisor should distribute and collect forms and turn in to Project Manager to have on file. These forms must be kept on file for seven years.

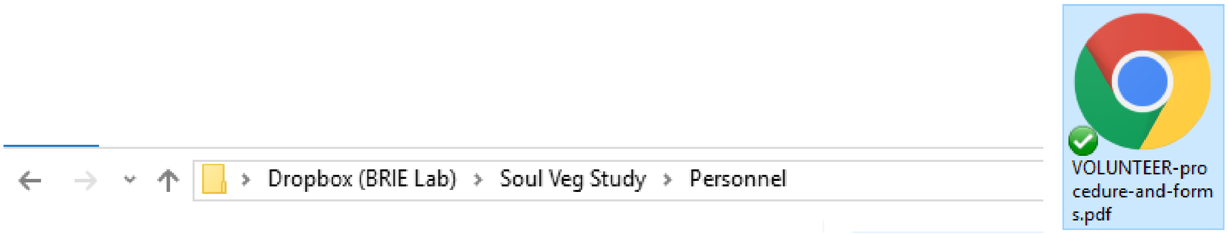

Staff and Volunteer Training

*Lab staff/volunteers must complete the following steps/trainings prior to working with study participants:

- Review guidelines for interacting with study participants
- In-person lab orientation/training with Project Manager (This training includes how to take accurate measurements, lab flow from participant check-in to check-out, emergency situations, proper attire, proper hygiene and data collection and tracking.)
- Register for eIRB: http://sc.edu/about/offices_and_divisions/research_compliance/irb/getting_started.php
  - Overview: http://sc.edu/about/offices_and_divisions/research_compliance/irb/training_and_education.php
- Complete below training. Email completion certificate to Mary Wilson mjwilson@mailbox.sc.edu
  - CITI Social & Behavioral Researchers & Social Behavioral Responsible Conduct of Research Training: https://about.citiprogram.org/en/course/human-subjects-research-2/

*Kitchen staff/volunteers must complete the following steps/trainings prior to working with study participants:

- Review guidelines for interacting with study participants
- In-person kitchen orientation/training with Nutrition Interventionist (This training includes knife skills for safe cutting, food safety, proper attire and proper hygiene.)

Incidents involving staff/volunteers- Students should be accompanied to the Student Health Center on campus by their direct supervisor or appointed core staff. If during a break and the Health Center is closed, students should be accompanied to the nearest urgent care/emergency room. Report the incident immediately to the Principal Investigator and Project Manager. As soon as possible document the incident and email a copy to the Principal Investigator and Project Manager as soon as possible.

Additional Forms for Student Staff/Volunteers

At the end of each semester, direct supervisors should share the Student RA form with each student and return to Project Manager via email. Additionally, *undergraduate* students who worked/volunteered with the study for 60 hours in any given semester, should register research experience through the university by following the steps below.

1. Visit https://sc.edu/about/offices_and_divisions/undergraduate_research/get-started/register_your_project/index.php
2. Carefully review the registration information on the above page.
3. Log into the Research Registry site using the username and password you use to access campus email: https://sc.edu/our/researchregistry/
4. Complete the form, answering all required prompts, and submit your research project information. List the staff person who you most closely work with as your mentor.

These forms can be found on dropbox in the personnel folder.

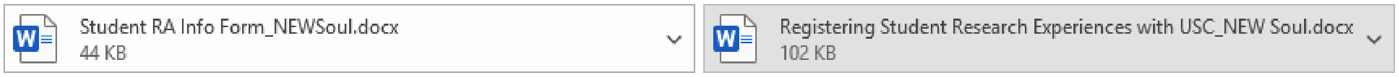

Helpful Information to Distribute to Student Staff & Volunteers

For those who do not have a car, we do not want you to be unsafe and walk alone in the dark. Please utilize on-campus services. See below screenshot and this link https://www.sc.edu/about/offices_and_divisions/parking/shuttles/safe_evening_rides/. Call the evening van ride or late-night shuttle and schedule your ride to and/or from work. See details below. As a last resort, you can also call USC Campus PD for an escort. Their dispatch number is 777-4215.

Late Night Shuttle

Phone: 803-777-3351
Shuttle Hours: 12:30 a.m. to 6:30 a.m.
Monday - Friday (while classes are in session)

- The shuttle will pick up USC student, faculty and staff from your location and take you anywhere you need to go on campus free of charge.
- You will be able to distinguish the shuttle by its white strobe light and a color scheme related to the Carolina Shuttle System.
- Wait in a secure area until it arrives.

Evening Van Rides

Phone: 803-777-3825
or call 803-777-3351 to reach the Late Night Shuttle if the van is not available.
Shuttle Hours: 8 p.m. to 12 midnight
Monday - Friday (while classes are in session)

- The Iota Mu Chapter of Alpha Phi Omega National Service Fraternity sponsors this free service to safely transport faculty, staff and students of USC.
- The van will pick you up and take you to any destination on campus.

GUIDELINES FOR DIETARY ADHERENCE CALLS

•

	CHECKLIST	NOTES
☐	After classes on Monday and Wednesday nights….. Collect and review all surveys completed in Monday and Wednesday classes.<br><br>For # 6., if participant responds with "0" or "1" days for consuming only foods meeting the NEW Soul Guidelines, put them in the stack of folks who need to be called.<br><br>For # 7., if participant responds with "Not at all" or "1-2days" for consuming FRUITS, VEGETABLES, LEGUMES/BEANS, WHOLE GRAINS, put them in the stack of folks who need to be called.<br><br>For #7, if participant is in the VEGAN group and responds that they consumed RED MEAT, POULTRY, FISH, EGGS, DIARY, FOODS COOKED IN OIL OR ADDED FAT 3-4 days or greater, put them in the stack of folks who need to be called.	PAGE 1 of SURVEY

WITH SOUL STUDY

Weekly Study Questionnaire

1. What is your participant ID number: ____________________
2. Diet Group (please circle): VEGAN / OMNI
3. What are your initials? __________________
4. Today's date: ______________
5. What is your current weight (in lbs)?: ______________________
6. Over the previous 7 days, how many days did you eat ***only*** foods meeting the NEW Soul Guidelines?

   0 1 2 3 4 5 6 7

7. How many days over the past 7 days did you consume these foods:

	Not at all	1-2 Days	3-4 Days	5-6 Days	Daily
Fruits	☐	☐	☐	☐	☐
Vegetables	☐	☐	☐	☐	☐
Legumes/Beans	☐	☐	☐	☐	☐
Whole grains	☐	☐	☐	☐	☐
Refined grains products like white bread, cookies, pastries or white flour crackers	☐	☐	☐	☐	☐
Red meat (beef or pork)	☐	☐	☐	☐	☐
Poultry	☐	☐	☐	☐	☐
Fish	☐	☐	☐	☐	☐
Eggs	☐	☐	☐	☐	☐
Dairy (cheese, yogurt, milk, etc.)	☐	☐	☐	☐	☐
Foods cooked in oil or added fat	☐	☐	☐	☐	☐

PLEASE TURN OVER AND COMPLETE THE BACK

1

	For #8, if participant responds that they consumed fast food that didn't meet the dietary program greater than 3 times in the last 7 days, put them in the stack of folks who need to be called.<br><br>For #13, if participant responds that they would like to speak to one of our team members for help, put them in the stack of folks who need to be called.	PAGE 2 of SURVEY<br><br>8. How many times over the last 7 days did you eat fast food that didn't meet your dietary program?<br>[ ] 1 time<br>[ ] 2 times<br>[ ] 3 times<br>[ ] 4 times<br>[ ] 5 times<br>[ ] more than 5 times<br><br>9. Which **weekday** meal was the **easiest** to follow with the NEW Soul Guidelines?<br>[ ] Breakfast<br>[ ] Lunch<br>[ ] Dinner<br>[ ] Snacks<br><br>10. Which **weekend** meal was the **easiest** to follow with the NEW Soul Guidelines?<br>[ ] Breakfast<br>[ ] Lunch<br>[ ] Dinner<br>[ ] Snacks<br><br>11. Which **weekday** meal was the **hardest** to follow with the NEW Soul Guidelines?<br>[ ] Breakfast<br>[ ] Lunch<br>[ ] Dinner<br>[ ] Snacks<br><br>12. Which **weekend** meal was the **hardest** to follow with the NEW Soul Guidelines?<br>[ ] Breakfast<br>[ ] Lunch<br>[ ] Dinner<br>[ ] Snacks<br><br>13. Would you like to speak to one of our members of our team about helping you with any diet-related challenges you're experiencing?<br>☐ Yes ☐ No
☐	Scan in the stack of responses who need to be called and email that file to Mary - mjwilson@mailbox.sc.edu.<br><br>The copier is located in the 5th floor mail room. The code is 2721	The remaining participant response who do not need to be called, should be filed in the participant's file behind the dietary adherence tab.
☐	Enter the responses of the participants who need to be called into the google spreadsheet.	You will need to access the database from on campus and enter the participant's phone number for the persons who will be calling. They will not have access to the database.<br><br>When you login to the database, click on "Participants" located on left hand side.<br>Scroll down to the bottom of the page and click "Search", enter the participant ID number and choose the dropbox tab of "all fields".

•

LITERATURE CITED

References

1. Fraser GE. Vegetarian diets: what do we know of their effects on common chronic diseases? *Am J Clin Nutr.* 2009;89(5):1607S-1612S.
2. Tonstad S, Stewart K, Oda K, Batech M, Herring RP, Fraser GE. Vegetarian diets and incidence of diabetes in the Adventist Health Study-2. *Nutr Metab Cardiovasc Dis.* 2013;23(4):292-299.
3. Barnard ND, Katcher HI, Jenkins DJ, Cohen J, Turner-McGrievy G. Vegetarian and vegan diets in type 2 diabetes management. *Nutr Rev.* 2009;67(5):255-263.
4. Key TJ, Davey GK, Appleby PN. Health benefits of a vegetarian diet. *Proc Nutr Soc.* 1999;58(2):271-275.
5. Craig WJ. Health effects of vegan diets. *Am J Clin Nutr.* 2009;89(5):1627S-1633S.
6. Glick-Bauer M, Yeh MC. The health advantage of a vegan diet: exploring the gut microbiota connection. *Nutrients.* 2014;6(11):4822-4838.
7. Barnard ND, Levin SM, Yokoyama Y. A Systematic Review and Meta-Analysis of Changes in Body Weight in Clinical Trials of Vegetarian Diets. *Journal of the Academy of Nutrition and Dietetics.* 2015;115(6):954-969.
8. Rizzo NS, Jaceldo-Siegl K, Sabate J, Fraser GE. Nutrient profiles of vegetarian and nonvegetarian dietary patterns. *J Acad Nutr Diet.* 2013;113(12):1610-1619.
9. Kaartinen K, Lammi K, Hypen M, Nenonen M, Hänninen O, Rauma A-L. Vegan diet alleviates fibromyalgia symptoms. *Scand J Rheumatol.* 2000;29(5):308-313.
10. Japas C, Knutsen S, Dehom S, Dos Santos H, Tonstad S. Body mass index gain between ages 20 and 40 years and lifestyle characteristics of men at ages 40–60 years: The Adventist Health Study-2. *Obes Res Clin Pract.* 2014;8(6):e549-e557.
11. Beezhold B, Radnitz C, Rinne A, DiMatteo J. Vegans report less stress and anxiety than omnivores. *Nutr Neurosci.* 2015;18(7):289-296.
12. Beezhold BL, Johnston CS, Daigle DR. Vegetarian diets are associated with healthy mood states: a cross-sectional study in seventh day adventist adults. *Nutr J.* 2010;9(1):1.
13. Dinu M, Abbate R, Gensini GF, Casini A, Sofi F. Vegetarian, vegan diets and multiple health outcomes: a systematic review with meta-analysis of observational studies. *Crit Rev Food Sci Nutr.* 2016;57(17):3640–3649.
14. Melby CL, Toohey ML, Cebrick J. Blood pressure and blood lipids among vegetarian, semivegetarian, and nonvegetarian African Americans. *The American journal of clinical nutrition.* 1994;59(1):103-109.
15. Oldways. African Heritage Diet Pyramid. 2011; https://oldwayspt.org/traditional-diets/african-heritage-diet. Accessed March 28, 2017.
16. Miller A. *Soul food: The surprising story of an American cuisine, one plate at a time.* UNC Press Books; 2013.
17. Cotton MA, Ball C, Robinson P. Four simple questions can help screen for eating disorders. *J Gen Intern Med.* 2003;18(1):53-56.
18. Seligman HK, Laraia BA, Kushel MB. Food insecurity is associated with chronic disease among low-income NHANES participants. *The Journal of nutrition.* 2010;140(2):304-310.
19. Sallis JF, Pinski RB, Grossman RM, Patterson TL, Nader PR. The development of self-efficacy scales for healthrelated diet and exercise behaviors. *Health Educ Res.* 1988;3(3):283-292.

20. Weller KE, Greene GW, Redding CA, et al. Development and validation of green eating behaviors, stage of change, decisional balance, and self-efficacy scales in college students. *Journal of nutrition education and behavior.* 2014;46(5):324-333.
21. Barnard ND, Gloede L, Cohen J, et al. A low-fat vegan diet elicits greater macronutrient changes, but is comparable in adherence and acceptability, compared with a more conventional diabetes diet among individuals with type 2 diabetes. *J Am Diet Assoc.* 2009;109(2):263-272.
22. Barnard ND, Scialli AR, Bertron P, Hurlock D, Edmonds K. Acceptability of a therapeutic low-fat, vegan diet in premenopausal women. *Journal of Nutrition Education.* 2000;32(6):314-319.
23. Barnard ND, Scialli AR, Turner-McGrievy G, Lanou AJ. Acceptability of a low-fat vegan diet compares favorably to a step II diet in a randomized, controlled trial. *J Cardiopulm Rehabil.* 2004;24(4):229-235.
24. Mason C, Katzmarzyk PT. Effect of the Site of Measurement of Waist Circumference on the Prevalence of the Metabolic Syndrome. *The American Journal of Cardiology.* 2009;103(12):1716-1720.
25. Ball SD, Altena TS, Swan PD. Comparison of anthropometry to DXA: a new prediction equation for men. *Eur J Clin Nutr.* 2004;58(11):1525-1531.
26. Pickering TG, Hall JE, Appel LJ, et al. Recommendations for Blood Pressure Measurement in Humans and Experimental Animals: Part 1: Blood Pressure Measurement in Humans: A Statement for Professionals From the Subcommittee of Professional and Public Education of the American Heart Association Council on High Blood Pressure Research. *Circulation.* 2005;111(5):697-716.
