# Supplementary material for: Effect of a Plant-Based vs Omnivorous Soul Food Diet on Weight and Lipid Levels Among African American Adults: A Randomized Clinical Trial
Source: JAMA Netw Open. 2023 Jan 12;6(1):e2250626. doi: 10.1001/jamanetworkopen.2022.50626 (PMC9857469; doi:10.1001/jamanetworkopen.2022.50626)
Supplement: Supplement 2. — Data Sharing Statement [file jamanetwopen-e2250626-s002.pdf]

Data Sharing Statement

Turner-McGrievy. Effect of a Plant-Based vs Omnivorous Soul Food Diet on Weight and Lipid Levels Among African American Adults. *JAMA Netw Open*. Published January 12, 2023. doi:10.1001/jamanetworkopen.2022.50626

Data
Data available: No

Additional Information
Explanation for why data not available: Once all papers from our group have been published from the data, we plan to release it for use by others.
